# Impact of sample size on the stability of risk scores from clinical prediction models: a case study in cardiovascular disease

**DOI:** 10.1186/s41512-020-00082-3

**Published:** 2020-09-09

**Authors:** Alexander Pate, Richard Emsley, Matthew Sperrin, Glen P. Martin, Tjeerd van Staa

**Affiliations:** 1 Centre for Health Informatics, School of Health Sciences, Faculty of Biology, Medicine and Health, The University of Manchester, Oxford Road, Manchester, M13 9PL UK; 2 Department of Biostatistics and Health Informatics, Institute of Psychiatry, Psychology and Neuroscience, King’s College London, De Crispigny Park, London, SE5 8AF UK

**Keywords:** Risk prediction, Sample size, Statistical methods, Precision, Stability

## Abstract

**Background:**

Stability of risk estimates from prediction models may be highly dependent on the sample size of the dataset available for model derivation. In this paper, we evaluate the stability of cardiovascular disease risk scores for individual patients when using different sample sizes for model derivation; such sample sizes include those similar to models recommended in the national guidelines, and those based on recently published sample size formula for prediction models.

**Methods:**

We mimicked the process of sampling *N* patients from a population to develop a risk prediction model by sampling patients from the Clinical Practice Research Datalink. A cardiovascular disease risk prediction model was developed on this sample and used to generate risk scores for an independent cohort of patients. This process was repeated 1000 times, giving a distribution of risks for each patient. *N* = 100,000, 50,000, 10,000, *N*_min_ (derived from sample size formula) and *N*_epv10_ (meets 10 events per predictor rule) were considered. The 5–95th percentile range of risks across these models was used to evaluate instability. Patients were grouped by a risk derived from a model developed on the entire population (population-derived risk) to summarise results.

**Results:**

For a sample size of 100,000, the median 5–95th percentile range of risks for patients across the 1000 models was 0.77%, 1.60%, 2.42% and 3.22% for patients with population-derived risks of 4–5%, 9–10%, 14–15% and 19–20% respectively; for *N* = 10,000, it was 2.49%, 5.23%, 7.92% and 10.59%, and for *N* using the formula-derived sample size, it was 6.79%, 14.41%, 21.89% and 29.21%. Restricting this analysis to models with high discrimination, good calibration or small mean absolute prediction error reduced the percentile range, but high levels of instability remained.

**Conclusions:**

Widely used cardiovascular disease risk prediction models suffer from high levels of instability induced by sampling variation. Many models will also suffer from overfitting (a closely linked concept), but at acceptable levels of overfitting, there may still be high levels of instability in individual risk. Stability of risk estimates should be a criterion when determining the minimum sample size to develop models.

## Background

Risk prediction models are used to guide clinical decision-making in a variety of disease areas and settings, ranging from the prevention of cardiovascular disease (CVD) in primary care to intensive care unit-based models such as APACHE or SOFA [1–5]. As such, developing risk prediction models appropriately is vital. One aspect of appropriate derivation of prediction models is ensuring sufficient sample size in the development dataset; unfortunately, sample size calculations for models are often not made, or at best are based on the simplistic “10 events per predictor” rule [6]. Risk prediction models that are recommended in treatment guidelines for routine use by clinicians are developed on cohorts of highly variable sizes. As an example, QRISK3 [7] (recommended by the National Institute for Health and Care Excellence to guide CVD primary prevention in England [8]) was developed on a cohort of 4,019,956 females and 3,869,847 males, whereas the pooled cohort equations (recommended by American College of Cardiology and American Heart Association to guide CVD prevention in the US [9]) were based on 9098 females and 11,240 males for white ethnicity, and 2641 females and 1647 males for African-American ethnicity.

If the sample size is too small, the most commonly cited issue is that of overfitting, which may result in over-optimistic model performance within the development dataset and poor model performance outside of the development dataset. Another potential issue, of which the implications are less clear, is that small sample sizes could lead to imprecise risk predictions. This means if a different cohort of individuals had been selected (at random) to develop the same model, resulting risk scores from the model may change significantly for a given individual. It is well known that differently defined prediction models may produce different risks for individuals, even if the models perform similarly on the population level (i.e. have similar performance metrics such as discrimination and calibration) [10–14]. This concept largely falls under the reference class problem [14], where a patient could be assigned multiple risk scores depending on which variables are adjusted for in the model, or assigned to different subgroups by stratifying on different variables. However, the variability in an individual’s risk score induced by using a small sample size is driven purely by statistical uncertainty, distinguishing this from the reference class problem.

The aim of this study was to evaluate the stability of CVD risk predictions for individual patients when using different sample sizes in the development of the risk prediction models, while also considering sample sizes from recent work focusing on overfitting and mean absolute prediction error (MAPE), representing state of the art techniques for sample size calculations in risk prediction models [15–17].

## Methods

### Data source

We defined two cohorts from a Clinical Practice Research Datalink (CPRD) [18] dataset, which comprised primary care data linked with Hospital Episode Statistics [19] (HES), and mortality data provided by the Office for National Statistics (ONS) [20]. For the first cohort (referred to as historical cohort), the cohort entry date was the latest of attaining age 25 years, attaining 1 year follow-up as a permanently registered patient in CPRD, or 1 Jan 1998. The end of follow-up was the earliest date of patient’s transfer out of the practice or death, last data collection for practice, or 31 Dec 2015. Patients were excluded if they had a CVD event (identified through CPRD, HES or ONS) or statin prescription prior to their cohort entry date (code lists available in additional file 1). The second cohort comprised patients actively registered on 1 Jan 2016 (referred to as contemporary cohort). This cohort of patients represents a contemporary population, for whom a risk prediction model would subsequently be applied to estimate their CVD risks. To be eligible for this second cohort, a patient had to be aged 25–85 years on 1 Jan 2016, and be actively registered in CPRD with 1 year prior follow-up with no history of CVD or statin treatment.

### Overview

We mimicked the process of sampling an overarching target population for the development of a risk prediction model by randomly sampling *N* patients from the historical cohort (containing 1,965,079 and 1,890,582 individuals for female and male cohorts respectively). A risk prediction model was developed on this sample and used to generate risk scores for the contemporary cohort. This process was repeated 1000 times, giving 1000 risk scores for each patient, for each sample size. The sample sizes considered were *N* = 10,000, 50,000, 100,000, *N*_epv10_ (sample size required to meet the 10 events per predictor rule) and *N*_min_ (minimum sample size required to meet criteria outlined by Riley et al. [15]). We chose 10,000 as it is similar to the number of females and males used to develop ASSIGN [21] (6540 and 6757), Framingham [22] (3969 and 4522) and Pooled Cohort Equations [9] (9098 and 11 240). The upper limit of 100,000 was chosen to match the SCORE [23] equations, which were developed on 117,098 and 88,080 females and males respectively. The criteria by Riley et al. [15] ensure that overfitting is minimised on both the relative scale (through the shrinkage factor) and the absolute scale (small difference between apparent and adjusted proportion of variance explained), and that the overall risk is estimated with a sufficient level of precision. Derivation of *N*_min_ = 1434 (female) and 1405 (male) and *N*_epv10_ = 2954 (female) and 2297 (male) is described in additional file 2. There are also sample size formula suggested by van Smeden et al. [17], which focus on minimising the MAPE or root mean squared prediction error (rMSPE) of the resulting model; however, the formula are for logistic models, so they could not be used in this study. Prediction error is closely linked to the variability in risk considered in this work (if risk scores are unbiased and there was little variability, then the MAPE and rMSPE would both be small). It was important to consider prediction error in this work, and the process for doing this is outlined later in the “Methods” section.

### Generation of risk scores

The historical cohort and contemporary cohort were both split into female and male cohorts, and missing data was imputed using one stochastic imputation using the mice package [24]. All variables included in QRISK3 [7], including the Nelson Aalen estimate of the baseline cumulative hazard at the event time and the outcome indicator, were included in the imputation process. The following process was then carried out separately for females and males: 100,000 individuals were chosen at random from the historical cohort to form an internal validation cohort, the remaining individuals formed the development cohort. The development cohort (containing 1,865,079 and 1,790,582 individuals for female and male cohorts respectively) was then viewed as the population.

First, we calculated a 10-year risk for each patient in the contemporary cohort and the validation cohort using a model developed on the entire development cohort, called the population-derived risks. To do this, a Cox model was fit to the development cohort, where the outcome was defined as the time until the first CVD event. Predictor variables included in the model were continuous variables, and categorical variables with > 1% prevalence in all categories calculated from the entire development cohort (age, body mass index, cholesterol/high density lipoprotein ratio, family history of CVD, treated hypertension, smoking status, systolic blood pressure, Townsend deprivation index and type 2 diabetes). These 9 variables resulted in 13 model coefficients. This set of variables reflects the smaller number of variables used in models with lower sample sizes in practice [9, 21, 22]. The risks were calculated by multiplying the cumulative baseline hazard of the model at 10 years follow-up, by the exponent of the linear predictor for each individual, and converting into a survival probability using standard survival analysis relationships. Harrell’s C [25] and the calibration-in-the-large (mean predicted risk − observed/Kaplan Meier risk) of this model were also calculated in the validation cohort. Calibration is reported on the % scale (as an absolute difference in risk), as opposed to probability scale.

**Fig. 1:**
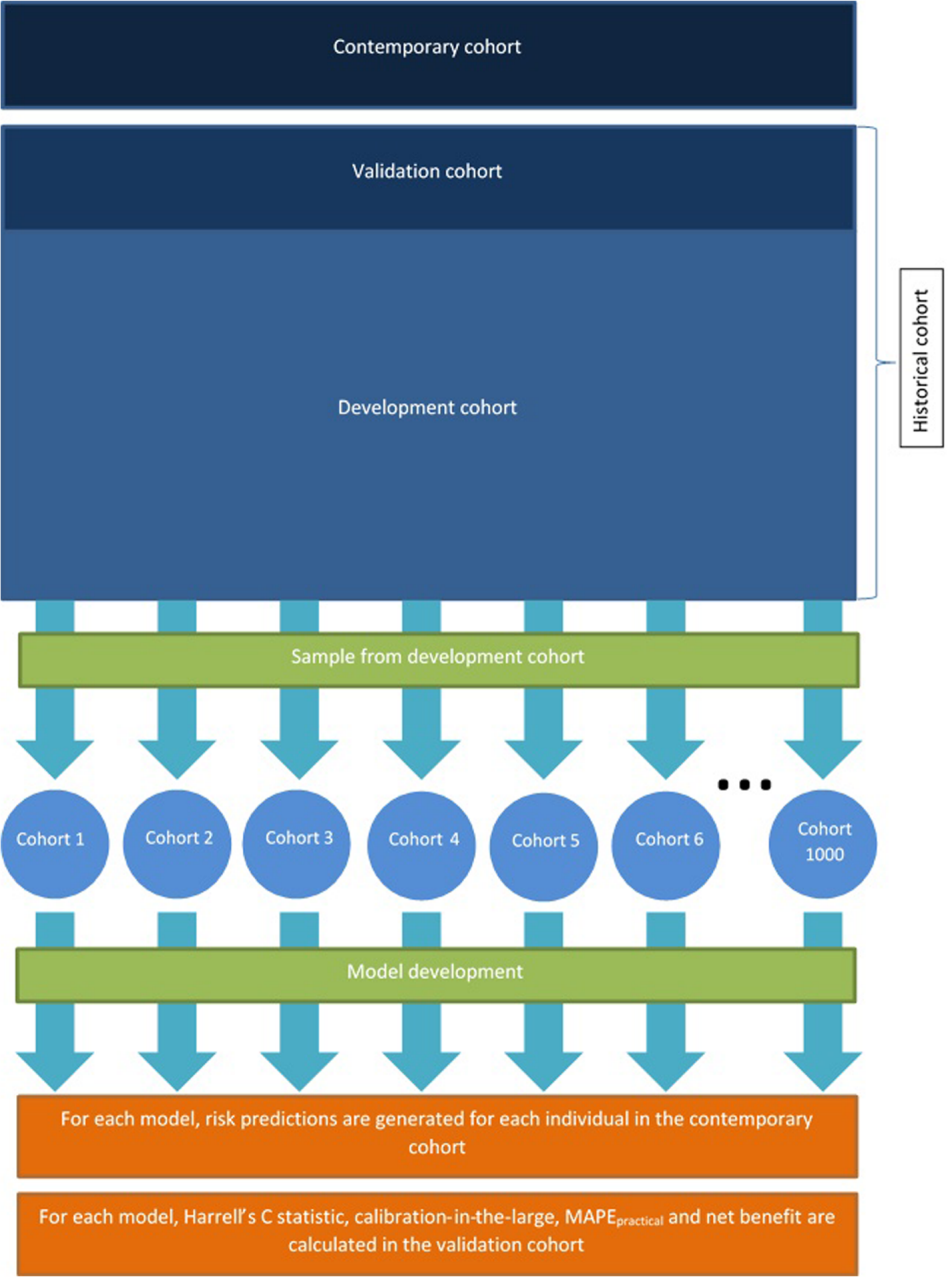
A graphical representation of the sampling process

Next, for each value of *N*, we sampled *N* patients from this population (the development cohort) without replacement, 1000 times. The following process was repeated within each sample. A Cox model was fit to the sampled data using the techniques described in the previous paragraph. The developed model was used to generate 10-year risk scores for each individual in the contemporary cohort and the validation cohort. Harrell’s C [25] statistic for this model and the calibration-in-the-large were calculated in the validation cohort. The mean absolute prediction error (MAPE_practical_) was also calculated for each model. This was the average (across patients) difference between the predicted risks and population-derived risks of patients in the validation cohort (difference calculated on the % scale, as opposed to probability). Note that we distinguish MAPE_practical_ from the MAPE used in the work by van Smeden et al. [17]. This is because in the present study, there is no “true” risk from which individual’s risk scores may deviate from and instead the population-derived risk is used. This can be thought of as a practical approximation to the MAPE metric used in the study by van Smeden et al. [17]. A graphical representation of the sampling process is given in Fig. 1.

### Analysis of stability of risk scores

For each sample size, the stability of risks for each patient in the contemporary cohort across the 1000 models was calculated in the following ways. First, the 5–95th percentile range of risks for each patient across the 1000 models was calculated. The distribution of these percentile ranges was then plotted in box plots stratified by the population-derived risk. Next, the 5–95th percentile range of risk for each patient was calculated across the subset of models with the highest C-statistic (top two thirds of models and top third of models). These percentile ranges were again presented in box plots stratified by population-derived risk. This process was repeated, restricting models to those where the calibration-in-the-large deviated from that of the population derived model the least (top two thirds of models and top third of models). This process was repeated again, restricting models to those where MAPE_practical_ was as small as possible (top two thirds of models and top third of models). This allowed us to explore whether only considering models with high discrimination, good calibration-in-the-large or small MAPE_practical_ would reduce the instability in the risk scores of individuals across these models. Finally, we grouped patients from the contemporary cohort into risk groups of width 1% as defined by their population-derived risk. The proportion of the 1000 models that classified a patient on the opposite side of the 10% risk threshold from the population-derived risk was then calculated (10% is threshold for statin eligibility according to the recommended guidelines in the UK [8]). This can be interpreted as the probability that an individual from a given risk group will be assigned a risk score on the opposite side of the treatment threshold, and highlights the impact this variability may have on an individual’s treatment decision in practice. For contrast, we also reported the net benefit [26, 27] of each model at the 10% threshold in the validation cohort, which informs on the impact this variability has on the population level.

Note that instability was assessed in the contemporary cohort as this cohort best represents the people who would have their risk assessed in practice today. Due to a lack of follow-up, model performance could not be assessed in the contemporary cohort. Instead, it was assessed in the same cohort the model was developed on (split sample internal validation), as would be done in practice if a dataset was not available for external validation.

## Results

The baseline characteristics for the female development cohort, validation cohort and the contemporary cohort are provided in Table 1. See additional file 3 for the equivalent table for the male cohort. There was missing data for ethnicity (57.93% and 58.16% for female and male cohorts respectively), BMI (31.17% and 46.38%), cholesterol/HDL ratio (61.52% and 64.29%), SBP (18.99% and 40.79%), SBP variability (49.61% and 79.06%) and smoking status (24.82% and 34.83%). Note that not all these variables were used to derive risk scores in this paper, but they were included in the imputation process to ensure imputed values were as accurate as possible.

**Table 1:** Baseline characteristics of each female cohort

		Development (*n* = 1,865,079)	Validation (*n* = 100,000)	Contemporary (*n* = 387,557)
Outcome	Total CVD events	82 065	4482	NA
	Total follow-up (years)	13 098 449	703 471	NA
Age		43.07 (15.94)	43.14 (15.96)	48.38 (14.43)
Systolic blood pressure		123.91 (18.28)	124 (18.22)	123.97 (15.17)
Body mass index		25.6 (5.60)	25.56 (5.56)	27.1 (6.31)
Cholesterol/high-density lipoprotein ratio		3.72 (1.20)	3.72 (1.21)	3.46 (1.04)
Smoking status	Never	56.04%	56.15%	46.05%
	Ex	16.97%	16.98%	31.66%
	Current	27.00%	26.87%	22.29%
Townsend	1 (least deprived)	21.96%	21.96%	24.95%
	2	21.99%	21.81%	22.35%
	3	21.17%	21.46%	21.56%
	4	20.46%	20.36%	18.70%
	5 (most deprived)	14.42%	14.41%	12.44%
Treated hypertension		6.18%	6.19%	8.45%
Family history of CVD		15.08%	15.13%	20.86%
Type 2 diabetes		1.16%	1.19%	1.15%

For continuous variables, the mean (standard deviation) is reported. There is no follow-up reported (NA) for the contemporary cohort because individuals entered the cohort on 1 Jan 2016, and follow-up in the CPRD extract stopped 3 months after this

The distribution of the C-statistic, calibration-in-the-large, MAPE_practical_ and net benefit of the 1000 models for each sample size are given in Table 2. The 97.5th percentile of C-statistic was similar for each sample size, but as the sample size decreased, the 2.5th percentile got smaller (0.802 vs 0.868 female and 0.805 vs 0.843 male). All C-statistic in the 2.5–97.5th percentile range were > 0.8. The variation in the calibration-in-the-large decreased as the sample size increased. The 2.5–97.5th percentile ranges of the calibration-in-the-large values were 2.61% (female) and 3.12% (male) for *N* = *N*_min_, decreasing to 0.32% (female) and 0.36% (male) for *N* = 100,000. Note that the calibration-in-the-large is not centred on zero, but we do not believe this affects the validity of the results. QRISK3 [7] suffers from a similarly poor calibration-in-the-large, yet is well calibrated within risk deciles. This is discussed further in the “Discussion” section. There was an improvement in the MAPE between the 2.5th and 97.5th percentile of the models as the sample size increased, ranging from 1.13% to 2.46% (female) and 1.34% to 2.91% (male) when *N* = *N*_min_, and from 0.13% to 0.28% (female) and 0.14% to 0.32% (male) when *N* = 100,000. There was also an improvement in the net benefit as sample size increased, ranging from 0.017 to 0.021 (female) and 0.024 to 0.029 (male) when *N* = *N*_min_, and from 0.021 to 0.022 (female) and 0.028 to 0.029 (male) when *N* = 100,000.

**Table 2:** Quantiles of C-statistic, calibration-in-the-large MAPE_practical_ and net benefit of the 1000 models, for each sample size

		Female					Male				
	Sample size	2.5%	25%	50%	75%	97.5%	2.5%	25%	50%	75%	97.5%
C-statistic	*N*_min_	0.802	0.852	0.857	0.861	0.864	0.805	0.827	0.831	0.835	0.839
	*N*_epv10_	0.856	0.861	0.863	0.865	0.867	0.826	0.834	0.837	0.839	0.841
	10,000	0.865	0.866	0.867	0.867	0.868	0.840	0.841	0.842	0.843	0.843
	50,000	0.867	0.868	0.868	0.868	0.868	0.843	0.843	0.843	0.843	0.844
	100,000	0.868	0.868	0.868	0.868	0.868	0.843	0.843	0.843	0.844	0.844
Calibration-in-the-large	*N*_min_	−2.22	−1.43	−0.95	−0.47	0.39	−2.56	−1.49	−1.01	−0.45	0.56
	*N*_epv10_	−1.85	−1.27	−0.97	−0.64	−0.11	−2.23	−1.47	−1.02	−0.60	0.29
	10,000	−1.45	−1.13	−0.95	−0.78	−0.44	−1.61	−1.20	−1.01	−0.80	−0.39
	50,000	−1.18	−1.03	−0.95	−0.87	−0.73	−1.28	−1.11	−1.02	−0.93	−0.77
	100,000	−1.11	−1.01	−0.96	−0.90	−0.79	−1.21	−1.08	−1.02	−0.95	−0.85
Mape_practical_	*N*_min_	1.13	1.53	1.75	2.00	2.46	1.34	1.79	2.04	2.34	2.91
	*N*_epv10_	0.76	1.03	1.20	1.36	1.74	1.00	1.36	1.57	1.78	2.26
	10,000	0.42	0.55	0.63	0.73	0.90	0.48	0.63	0.73	0.85	1.04
	50,000	0.19	0.25	0.28	0.32	0.40	0.21	0.29	0.33	0.37	0.45
	100,000	0.13	0.17	0.20	0.22	0.28	0.14	0.20	0.23	0.26	0.32
Net benefit	*N*_min_	0.017	0.019	0.020	0.021	0.021	0.024	0.026	0.027	0.028	0.029
	*N*_epv10_	0.020	0.021	0.021	0.021	0.022	0.026	0.027	0.028	0.028	0.029
	10,000	0.021	0.021	0.021	0.022	0.022	0.028	0.028	0.028	0.029	0.029
	50,000	0.021	0.022	0.022	0.022	0.022	0.028	0.029	0.029	0.029	0.029
	100,000	0.021	0.022	0.022	0.022	0.022	0.028	0.029	0.029	0.029	0.029

Performance metrics of the population derived models were as follows. C-statistic: 0.868 (female) and 0.844 (male). Calibration-in-the-large: −0.95% (female) and −1.02% (male). Net benefit: 0.022 (female) and 0.029 (male)

**Fig. 2:**
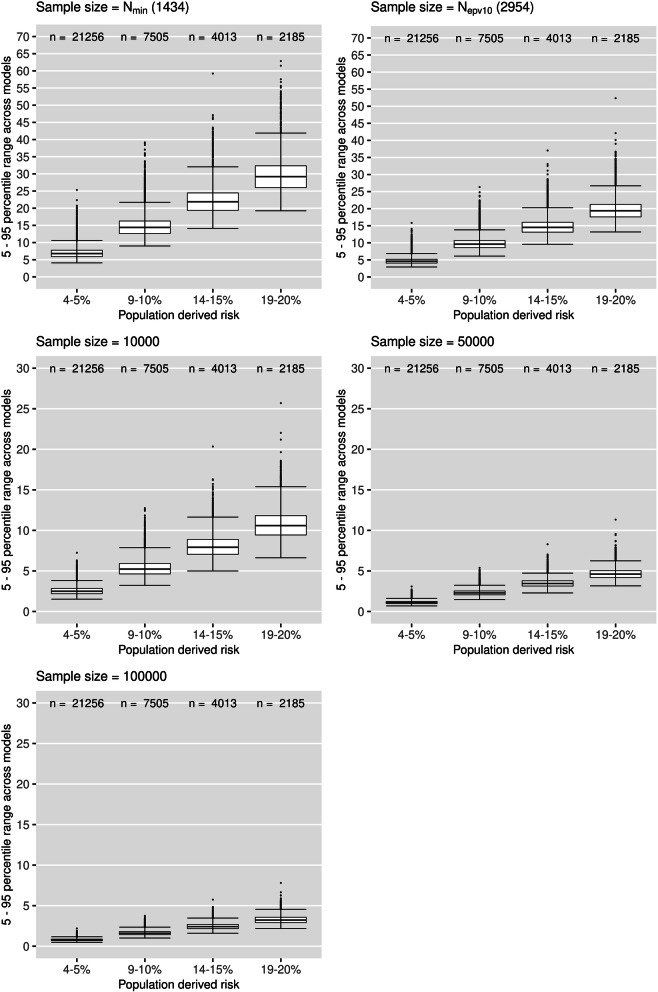
Boxplots of the percentile ranges of risk for individuals across the 1000 models (female cohort). Each data point represents the 5–95th percentile range in risk for an individual across the 1000 models

Figure 2 plots the 5–95th percentile range in risks for patients across the 1000 models, grouped by population-derived risk (female cohort). Specifically, each data point making up the boxplots is the 5–95th percentile range in risk across the 1000 models for an individual. The box plots are done in Tukey’s style [28], where outliers are plotted separately if they are more than 1.5 times the interquartile range below or above the 25th and 75th percentiles respectively. Note that these limits on the boxplot are distinct from the 5–95th percentile range in risk for each individual. The number of patients contributing to each box plot (defined by the population-derived risk) is stated at the top of the graph. For *N* = 100,000, the median 5–95th percentile range was 0.77%, 1.60%, 2.42% and 3.22% for patients in the 4–5%, 9–10%, 14–15% and 19–20% risk groups respectively. For *N* = 50,000, the median percentile range was 1.10%, 2.29%, 3.45% and 4.61% in the respective groups; for *N* = 10,000, it was 2.49%, 5.23%, 7.92% and 10.59%; for *N* = *N*_epv10_, it was 4.60%, 9.61%, 14.52% and 19.39%; and for *N* = *N*_min_, it was 6.79%, 14.41%, 21.89% and 29.21%. For each sample size, there was a linear relationship between the median percentile range of each group and the population-derived risk of that group. For example, for a sample size of 10,000, the median percentile range was always approximately 50% of the population-derived risk. For *N*_min_, the median percentile range was always approximately 150% of the population-derived risk. Results for the male cohort followed a similar pattern, but the level of instability was slightly lower (additional file 3).

**Fig. 3:**
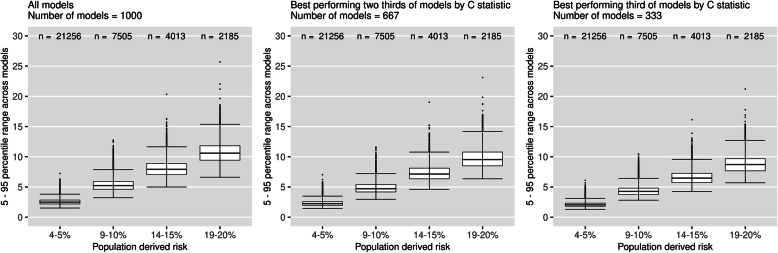
Percentile ranges of risk for individuals, subsetted by C-statistic of the models (female cohort, *N* = 10,000). Each data point represents the 5–95th percentile range in risk for an individual across a group models defined by their C-statistic. Two thirds of models had C-statistic > 0.866%, and one third of models had C-statistic > 0.867%

Figure 3 plots the 5–95th percentile range in risks for patients across models subsetted by the C-statistic of the models (female cohort, *N* = 10,000). The median 5–95th percentile range across models with C-statistic in the top third was 2.05%, 4.27%, 6.47% and 8.71% for patients in the respective risk groups. This equates to an 18–19% reduction in the median percentile range when using well discriminating models compared to all models (2.49%, 5.23%, 7.92% and 10.59%). Results for other sample sizes presented in additional file 3.

**Fig. 4:**
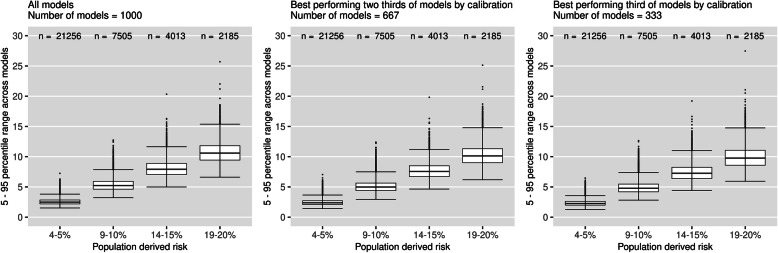
Percentile ranges of risk for individuals, subsetted by calibration-in-the-large of the models (female cohort, *N* = 10,000). Each data point represents the 5–95th percentile range in risk for an individual across a group models defined by their calibration-in-the-large. Two thirds of models had calibration-in-the-large within 0.25% of the population-derived model, one third of models had calibration-in-the-large within 0.14% of the population-derived model

Figure 4 plots the 5–95th percentile range in risks for patients across models subsetted by the calibration-in-the-large of the models (female cohort, *N* = 10,000). The median 5–95th percentile range across models with the best calibration-in-the-large was 2.29%, 4.78%, 7.26% and 9.77%, for the respective risk groups. This equates to a 8–9% reduction in the median percentile range compared to when using all models (2.49%, 5.23%, 7.92% and 10.59%). Results for other sample sizes presented in additional file 3.

**Fig. 5:**
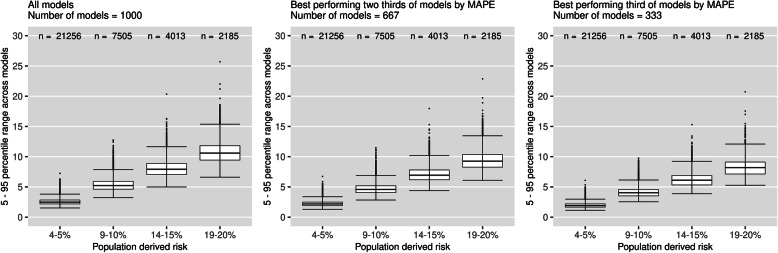
Percentile ranges of risk for individuals, subsetted by the MAPE_practical_ of the models (female cohort, *N* = 10000). Each data point represents the 5–95th percentile range in risk for an individual across a group models defined by their MAPE_practical_ score. Two thirds of models had MAPE_practical_ < 0.69%, and one third of models had MAPE_practical_ < 0.58%

Figure 5 plots the 5–95th percentile range in risks for patients across models subsetted by the MAPE_practical_ of the models (female cohort, *N* = 10,000). The median 5–95th percentile range across models with the MAPE_practical_ in the top third was 1.92%, 4.04%, 6.11% and 8.20%, for the respective risk groups. This equates to a 23% reduction in the median percentile range compared to when using all models (2.49%, 5.23%, 7.92% and 10.59%). Results for other sample sizes presented in additional file 3.

**Fig. 6:**
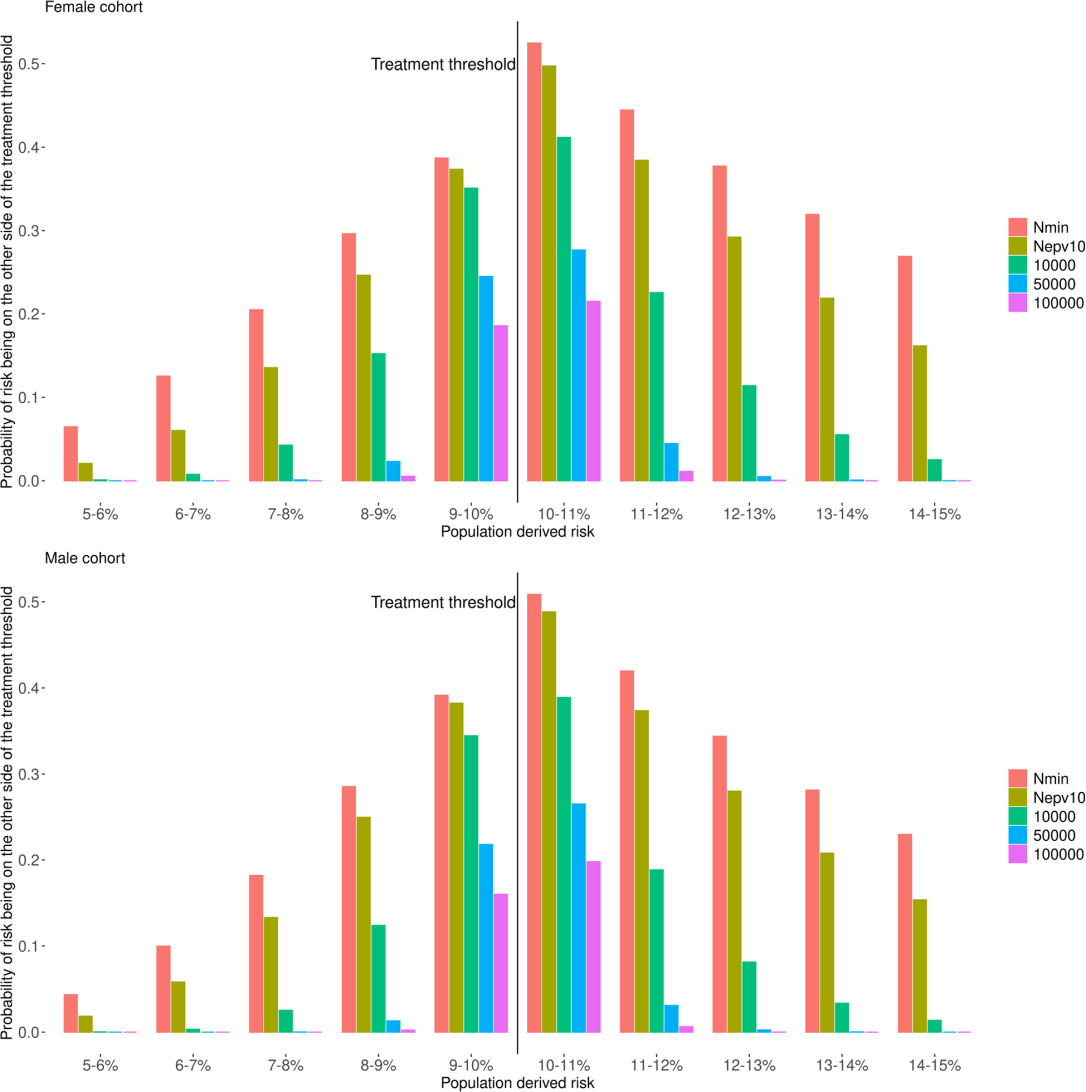
Probability of being classified on the opposite side of the 10% risk treatment threshold. This is the probability that a patient from a specified given population-derived risk group would be classified on the opposite side of the 10% treatment threshold by a randomly selected model

Figure 6 shows the probability that a patient from a given risk group (according to population derived model) may be classified on the opposite side of the 10% threshold by a randomly chosen model. For example, when using a sample size of *N*_min_, 26.91% of patients with a population-derived risk between 14 and 15% would be classified as having a risk below 10%; for *N* = *N*_epv10_, it would be 16.18%, whereas this is only 2.50% for *N* = 10,000, 0.01% for 50,000 and < 0.01% for 100,000.

## Discussion

This study found that at sample sizes typically used for developing risk models (e.g. in the CVD domain, the pooled cohort equations [9] and ASSIGN [21] were based on approximately 10,000 individuals or less), there is substantial instability in risk estimates attributable to sampling variability. Furthermore, when restricting the analysis to models with discrimination, calibration or MAPE in the best-performing third, high levels of instability remained across these models.

This variability in individual risk is especially relevant if using the model to make clinical decisions based on whether a risk score is above or below a fixed threshold (a common use for risk prediction models). From an individual’s and clinician’s perspective, it is unsatisfactory that a different treatment decision may be made depending on the model used. However, this is also an issue at the population level. Consider statin therapy in the UK. Initiating statins in patients who have a 10-year risk of CVD > 10% has been shown to be cost effective [29]. This intervention becomes more cost effective the better the performance (calibration and discrimination) of the model used to calculate the risk scores. Sample size is correlated with model performance, and a small sample size will likely lead to a worse performing model, and a smaller net benefit of the model (as found in Table 2).

Unfortunately, it is difficult to assess when increasing sample size will improve model performance, given that model performance is affected by many other factors (prevalence of outcome, inclusion of important predictors, strength of association between predictors and outcome) [30, 31]. Sample size affects model performance through the precision of coefficients. Imprecise estimates increase the probability that the risk of subgroups (a group of individual’s sharing the same set of covariates) in the population are miscalculated. Therefore, if the coefficients are precise, and risk estimates are stable, one will not be able to improve model performance by increasing the sample size further. This is unless increasing the sample size allows for extra predictors to be included without suffering from overfitting. Therefore, the stability of risk scores (and ultimately precision of coefficients) could be used as a proxy to determine whether increasing sample size will improve model performance (assuming the set of predictor variables is fixed). When *N* = 10,000, we see levels of instability that indicate the performance of the model could be improved by increasing sample size, resulting in fewer CVD events. However, in practice, the cost of recruitment of extra patients may have to be weighed up against the potential gain in net benefit.

At the minimum sample size suggested by Riley et al. [15], the instability in risk is even higher and the issues are heightened. There are no CVD risk prediction models used in practice that are developed on cohorts with sample sizes this small as there is often ample data to produce CVD risk prediction models. However, this may not be the case for other disease areas, where the outcomes are not well recorded in routinely collected datasets. In this scenario, one may have to actively recruit patients into a cohort and the work by Riley et al. [15] may be used in order to derive a sample size as opposed to the events per variable = 10 rule. We propose that if risk scores from a model are going to be used to drive clinical decision-making above or below a fixed threshold, section 6 of Riley et al. [15], “Potential additional criterion: precise estimates of predictor effects” should be properly considered. It is imprecise estimates of the predictor effects that lead to instability of risk scores. If this criterion is not met, as is the case for *N* = *N*_min_ in this paper, risk scores have high levels of instability and models will likely have poorer performance. The number of patients required to ensure stable risk scores will depend on the prevalence of the outcome, the number of predictors and the strength of the association between outcomes and predictors among other things, and therefore will vary for each model.

Increasing the sample size was associated with lower MAPE_practical_ in the resulting models (Table 2), and restricting to models with a small MAPE had a bigger impact on instability in risk than calibration or discrimination (although it is difficult to directly compare Figs. 3, 4 and 5). Therefore, a sample size formula based on the MAPE may be useful for Cox risk prediction models. However, more thought needs to be given to the cut-off as to what is acceptable. In recent sample size guidelines for logistic risk prediction models [16], a MAPE of no larger than 0.05 is suggested (corresponding to an average absolute error of 5% in predicted risk). In this study, when *N* = *N*_min_, we found very high levels of instability despite the MAPE_practical_ being much smaller than 5%, so a smaller cut-off may have to be used in practice. Alternatively, our work provides a way to ensure prediction error is below a certain level for individuals of a given risk, as opposed to the average prediction error over all patients which may be heavily dependent on a large number of lower-risk individuals. This is also advantageous as it avoids emphasis on error from an unobservable “true” risk, instead viewing this as variability (over sampling) in the predicted risk for an individual.

In practice, to ascertain whether a given development cohort has a sufficient sample size, the process undertaken in this manuscript could be replicated using bootstrap resampling methods. Instead of sampling the population without replacement (not possible in practice), sampling the development cohort with replacement (i.e. bootstrapping) can replicate this process and one could obtain a similar range of risks for each patient by fitting models on each of the bootstrapped samples. While the risks would be centred on the risk of that sample rather than the population-derived risk, the level of variation would be the same, meaning the stability of the risk scores could still be assessed. A decision could then be made on whether more patients should be recruited. One proposal on how to use this information to determine a sufficient sample size could be to ensure the bootstrapped 5–95th percentile range for all patients must be smaller than *x*% of their estimated risk. Another proposal may be to ensure that, for patients whose estimates are a certain distance away from a treatment threshold, there is a less than an *x*% chance of deriving a risk on the other side of the treatment threshold. This would mean that if the population was resampled and a model was developed on this new cohort, there would be less than an *x*% chance of the treatment pathway changing for that individual.

There are some limitations that warrant discussion. The first is that the calibration-in-the-large of the population-derived model was poor. We do not believe this is a problem as a similar miscalibration-in-the-large is found in QRISK3 [7], despite the model being well calibrated within risk deciles. It is likely caused by incompatible assumptions under how the observed risks (Kaplan-Meier assumes unconditional independent censoring) and predicted risks (Cox model assumes independent censoring only after conditioning on the covariates) are estimated. When looking within risk deciles, the difference in assumptions is not as large and good calibration was found. Centering the calibration-in-the-large measurements thus allowed the evaluation of whether the instability in risk was being driven by over- and under-predicting models. A second limitation was that this study concerned the outcome CVD and used a specific set of variables for prediction, rather than carrying out a systematic simulation study. This means we were unable to explore what specific aspects of the model development process may be driving the uncertainty (for example, the factors mentioned before, such as the prevalence of the outcome, the predictors used and the strength of the association between the outcomes and predictors). This means the results are directly applicable to CVD risk prediction, but generalisability of the other disease areas is limited, and similar studies to this one should be carried out in these disease areas.

As an area for future research, we would like to consider the impact that sampling variation may have on empirical choices about modelling structure (i.e. which variables are included when performing variable selection, what interaction terms are included or what the optimal functional form of continuous variables is). This paper focused solely on the direct impact of sampling variation, and the impact of these subsequent decisions on the instability of the resulting risk scores is not clear.

## Conclusions

In conclusion, CVD risk prediction models developed on randomly sampled cohorts of size 10,000 or less suffer from high levels of instability in individual risk predictions. There are multiple models used in practice that are developed on sample sizes this small. To avoid this, models should be developed on larger cohorts such as the QRISK3 [7] and SCORE [23] models. More generally, if developing a risk prediction model to guide treatment for patients above a fixed threshold, consideration should be given to the stability of risk scores and precision of effect estimates when choosing a sample size.

## Supplementary information

**Additional file 1.** Predictor variable information and code lists. More detailed information on how variables were extracted from the electronic health record to be used for analysis, including code lists

**Additional file 2.** Calculation of Nmin. Calculation of the minimum required sample size according to published sample size formula references in the manuscript. Separate calculations for male and female cohorts

**Additional file 3.** Supplementary tables and figures. Baseline demographics of male cohorts and results from simulations that could not be included in the main manuscript for space reasons

## Data Availability

The datasets generated and/or analysed during the current study are not publicly available as this would be a breach of the contract with CPRD. However, it can be obtained by a separate application to CPRD after getting approval from Independent Scientific Advisory Committee (ISAC). To apply for data, follow the instructions here: https://www.cprd.com/research-applications. The code used for all analyses is provided in a reusable format at the following GitHub page: https://github.com/alexpate30/Impact-of-sample-size-of-the-stability-of-risk-scores. Simulated data is also provided to run the code on and produce dummy figures and tables. Full details on how to implement the code is provided on the GitHub page.

## Abbreviations

CVD: Cardiovascular disease
CPRD: Clinical Practice Research Datalink
HES: Hospital Episode Statistics
ONS: Office for National Statistics
