# Supplementary material for: Impact of sample size on the stability of risk scores from clinical prediction models: a case study in cardiovascular disease
Source: Diagn Progn Res. 2020 Sep 9;4:14. doi: 10.1186/s41512-020-00082-3 (PMC7487849; doi:10.1186/s41512-020-00082-3)
Supplement: Supplementary file 2 — Additional file 2. Calculation of Nmin. Calculation of the minimum required sample size according to published sample size formula references in the manuscript. Separate calculations for male and female cohorts [file 41512_2020_82_MOESM2_ESM.pdf]

Additional file 2 – Calculation of N_{min}, minimum required sample size following published criteria, and N_{epv10}, sample size meeting the 10 events per predictor rule

Calculation of N_{min}

Methods

We followed Riley et al.'s[1] criteria for calculating the minimum required sample size for a risk prediction model. These are (i) ensure a global shrinkage factor $S_{VH} > 0.9$; (ii) ensuring a small absolute difference in the apparent and adjusted $R^2_{Nagelkerke}$; (iii) ensure precise estimate of overall risk (model intercept). The recommended estimate of the global shrinkage factor is the shrinkage factor of Van Houwelingen and Le Cessie[2], S_{VH}. The entity $R^2_{Nagelkerke}$[3] is an estimate of the proportion of variance explained, that always lies between 0 and 1. When estimating this quantity, the apparent estimate will be optimistic, and can be adjusted to get an unbiased estimate, which are the two values of interest here.

The main challenge in following these recommendations is the need to calculate $R^2_{CS\_ADJ}$[4] (an unbiased estimate of the Cox-Snell[5] R^2) to calculate the sample size, which can only be calculated after fitting the model. It is recommended to use metrics provided by previous prediction models developed on similar populations to estimate $R^2_{CS\_ADJ}$. In this study, we can use the model developed on the whole development cohort to calculate $R^2_{CS\_ADJ}$ directly. This value of $R^2_{CS\_ADJ}$ allows us to calculate the minimum required sample size for a model developed in this population.

Results

Calculation of N_{min}

We illustrate the steps for calculating N_{min} for the female cohort, following the process outlined in Riley et al.[1]

Criteria (i)

We start by calculating:

$$R^2_{CS\_APP} = 1 - \exp\left(\frac{LR}{n}\right)$$
$$= 0.0780$$

Where $R^2_{CS\_APP}$ is a biased estimate of the Cox-Snell[5] R^2 (based on the work by Magee[6]) , LR = likelihood ratio of the model developed on the entire population, and n = 1,865,078 is the size of the cohort used in that model. Next we calculate:

$$S_{VH} = 1 + \left(\frac{p}{n * \ln(1 - R^2_{CS_{APP}})}\right)$$
$$= 0.99991$$

Where S_{VH} is the global shrinkage factor of Van Houwelingen and Le Cassie[2], and p = 13 is the number of predictor variables. There are 9 variables, and but Smoking contributes two dummy variables (categories = yes/ex/never) and Townsend contributes 4 dummy variables (5 deprivation categories). Then we can calculate:

$$R^2_{CS\_ADJ} = S_{VH} * R^2_{CS\_APP}$$
$$= 0.0780$$

To get a model which has a shrinkage of at least S_{VH} = 0.9, as is recommended in the guidelines, we use the following formula:

$$N_{min} = \frac{p}{(S_{VH} - 1) * ln\left(1 - {R^2_{CS\_ADJ}}/{S_{VH}}\right)}$$
$$= \frac{13}{(0.9 - 1) * ln(1 - {0.0781}/{0.9})}$$
$$= 1434$$

Criteria (ii)

In order for the difference between the apparent and adjusted $R^2_{Nagelkerke}$[3] to be suitable, the following equation must be satisfied:

$$S_{VH} \geq \frac{R^2_{CS\_ADJ}}{R^2_{CS\_ADJ} + \delta * max\left(R^2_{CS\_APP}\right)}$$

Where $S_{VH} = 0.9$ is the desired shrinkage, δ is the acceptable difference between the apparent and adjusted $R^2_{Nagelkerke}$, and

$$max\left(R^2_{CS\_APP}\right) = 1 - exp\left(\frac{2 * \ln(L_{null})}{n}\right)$$
$$= 0.6987$$

where L_{null} is the log likelihood of the null model with no covariates, and was calculated directly from the population derived model. The recommended $\delta = 0.05$ and therefore:

$$\frac{R^2_{CS\_ADJ}}{R^2_{CS\_ADJ} + \delta * max\left(R^2_{CS\_APP}\right)} = \frac{0.0780}{0.0780 + 0.05 * 0.6987}$$
$$= 0.6906$$
$$\leq 0.9$$

and the criteria is satisfied.

Criteria (iii)

This requires that the confidence interval around the cumulative incidence at t, time point of interest, to be smaller than 0.05. We will assume an exponential distribution which is the simplest approach to this.

Let T = total follow up time in years if N_{min} is the sample size (average follow up multiplied by sample size), $\hat{\lambda}$ be the estimated number of events per person year, and t = 10 years is the point of interest (time at which we are making risk predictions). Then the confidence interval is then calculated as:

$$
\begin{aligned}
CI &= 1 - exp\left(-\left(\hat{\lambda} \pm 1.96 * \sqrt{\frac{\hat{\lambda}}{T}}\right) * t\right) \\
&= 1 - exp\left(-\left(0.0063 \pm 1.96 * \sqrt{\frac{0.0063}{7.0230 * 1434}}\right) * 10\right) \\
&= (0.0290, 0.0751)
\end{aligned}
$$

The size of the confidence interval is $0.0290 < 0.05$.

Therefore the value of N_{min} = 1434 satisfies all the criteria and is included as a sample size in our main analysis.

The exact same process was followed for the male cohort, and the value of N_{min} = 1405 was found to satisfy all the criteria.

Calculating of N_{epv10}

Female cohort

There are 13 coefficients, meaning a 130 events are required to meet the 10 events per predictor rule. There are 82 065 events in the development cohort of size 1 865 079. This means there are 0.0440 events per person, and 2 954 individuals are required (on average) to obtain 130 events.

Male cohort

There are 13 coefficients, meaning a 130 events are required to meet the 10 events per predictor rule. There are 101 360 events in the development cohort of size 1 790 582. This means there are 0.0566 events per person, and 2 296 individuals are required (on average) to obtain 130 events.

References

1. Riley RD, Snell KIE, Ensor J, et al. Minimum sample size for developing a multivariable prediction model: PART II - binary and time-to-event outcomes. *Stat Med* 2019; 38: 1276–1296.

2. Van Houwelingen J, Le Cessie S. Predictive value of statistical models. *Stat Med* 1990; 9: 1303–1325.

3. Nagelkerke NJD. A note on a general definition of the coefficient of determination. *Biometrika* 1991; 78: 691–692.

4. Mittlboeck M, Heinzl H. Pseudo R-squared measures of generalized linear models. In: *Proceedings of the 1st European Workshop on the Assessment of Diagnostic Performance*.

Milan, Italy, 2004.

5. Cox DR, Snell EJ. *Analysis of Binary Data*. 2 edition. Chapman and Hall/CRC, 1989.

6. Magee L. R2 measures based on wald and likelihood ratio joint significance tests. *Am Stat* 1990; 44: 250–253.
