# Supplementary material for: Impact of sample size on the stability of risk scores from clinical prediction models: a case study in cardiovascular disease
Source: Diagn Progn Res. 2020 Sep 9;4:14. doi: 10.1186/s41512-020-00082-3 (PMC7487849; doi:10.1186/s41512-020-00082-3)

**Additional file 3 – Supplementary tables and figures**

Table of Contents

[Supplementary Table 1: Baseline characteristics of each male cohort 2](#_Toc42165620)

[Supplementary Figure 1: Boxplots of the 5 - 95 percentile ranges in risk for individuals across the 1000 models (male cohort) 3](#_Toc42165621)

[Supplementary Figure 2: Boxplots of the 5 - 95 percentile ranges in risk for individuals across the models, stratified by the C statistic of the models (female cohort) 4](#_Toc42165622)

[Supplementary Figure 3: Boxplots of the 5 - 95 percentile ranges in risk for individuals across the models, stratified by the C statistic of the models (male cohort) 5](#_Toc42165623)

[Supplementary Figure 4: Boxplots of the 5 - 95 percentile ranges in risk for individuals across the models, stratified by the calibration-in-the-large of the models (female cohort) 7](#_Toc42165624)

[Supplementary Figure 5: Boxplots of the 5 - 95 percentile ranges in risk for individuals across the models, stratified by the calibration-in-the-large of the models (male cohort) 8](#_Toc42165625)

[Supplementary Figure 6: Boxplots of the 5 - 95 percentile ranges in risk for individuals across the models, stratified by the MAPE_practical_ of the models (female cohort) 10](#_Toc42165626)

[Supplementary Figure 7: Boxplots of the 5 - 95 percentile ranges in risk for individuals across the models, stratified by the MAPE_practical_ of the models (male cohort) 11](#_Toc42165627)

# Supplementary Table 1: Baseline characteristics of each male cohort

|  |  | Development  (n=1 790 582) | Validation  (n = 100 000) | Contemporary  (n = 352 026) |
| --- | --- | --- | --- | --- |
| Outcome | Total CVD events | 101360 | 5691 | NA |
|  | Total follow up (years) | 12291619 | 685616 | NA |
| Age |  | 41.85 (14.57) | 41.84 (14.53) | 46.86 (13.64) |
| Systolic blood pressure |  | 130.04 (16.48) | 129.89 (16.48) | 130.22 (13.93) |
| Body mass index |  | 26.12 (4.53) | 26.13 (4.55) | 27.43 (5.07) |
| Cholesterol/high density lipoprotein ratio |  | 4.48 (1.4) | 4.48 (1.38) | 4.17 (1.25) |
| Smoking status | Never | 46.63% | 46.55% | 37.17% |
|  | Ex | 17.37% | 17.52% | 30.07% |
|  | Current | 35.99% | 35.93% | 32.76% |
| Townsend | 1 (least deprived) | 21.65% | 21.56% | 24.18% |
|  | 2 | 21.50% | 21.46% | 21.74% |
|  | 3 | 20.77% | 20.89% | 21.61% |
|  | 4 | 20.77% | 20.94% | 19.03% |
|  | 5 (most deprived) | 15.30% | 15.15% | 13.45% |
| Treated hypertension |  | 4.51% | 4.45% | 7.16% |
| Family history of CVD |  | 11.02% | 11.00% | 14.85% |
| Type 2 diabetes |  | 1.42% | 1.43% | 1.46% |

# Supplementary Figure 1: Boxplots of the 5 - 95 percentile ranges in risk for individuals across the 1000 models (male cohort)


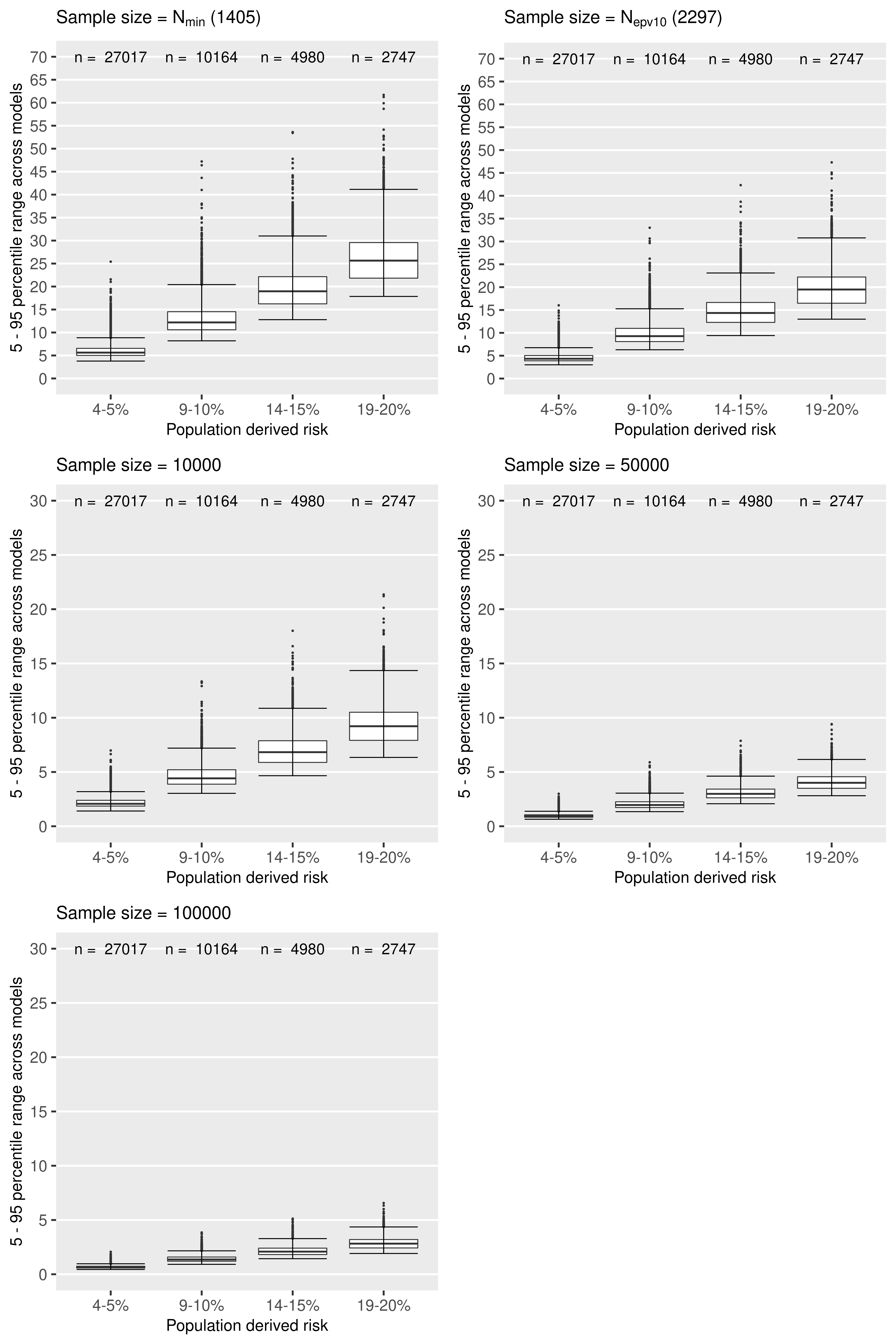


# Supplementary Figure 2: Boxplots of the 5 - 95 percentile ranges in risk for individuals across the models, subsetted by the C statistic of the models (female cohort)

Sample size = N_min_ (1434)


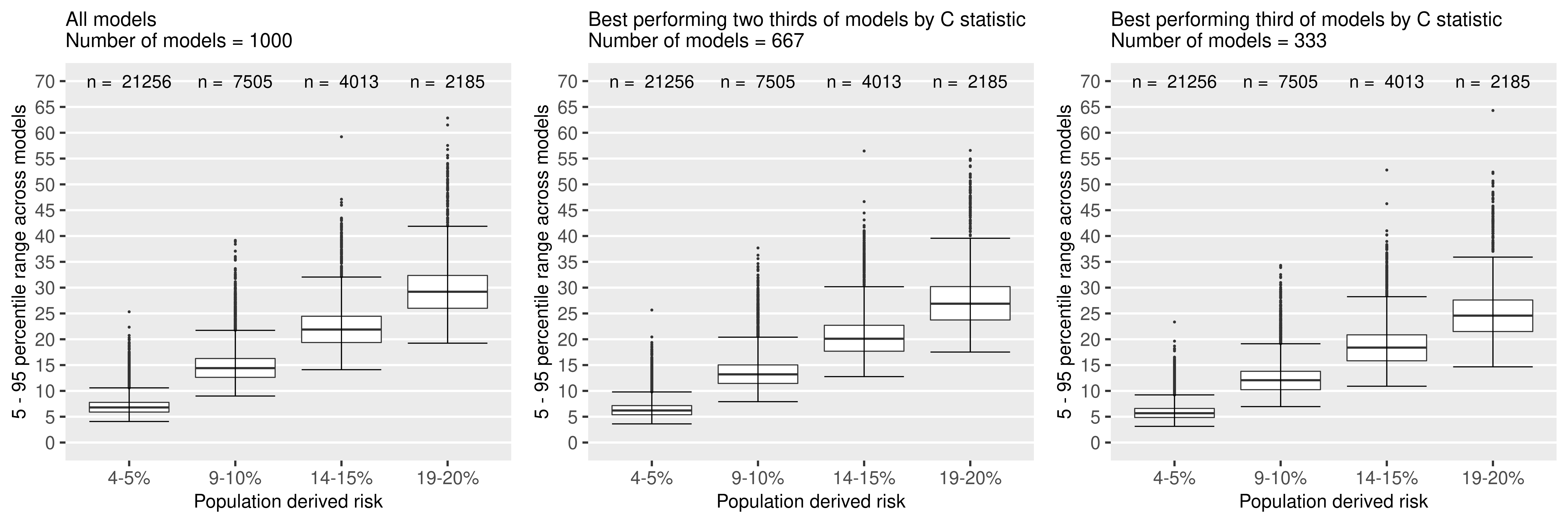


Sample size = N_epv10_ (2954)


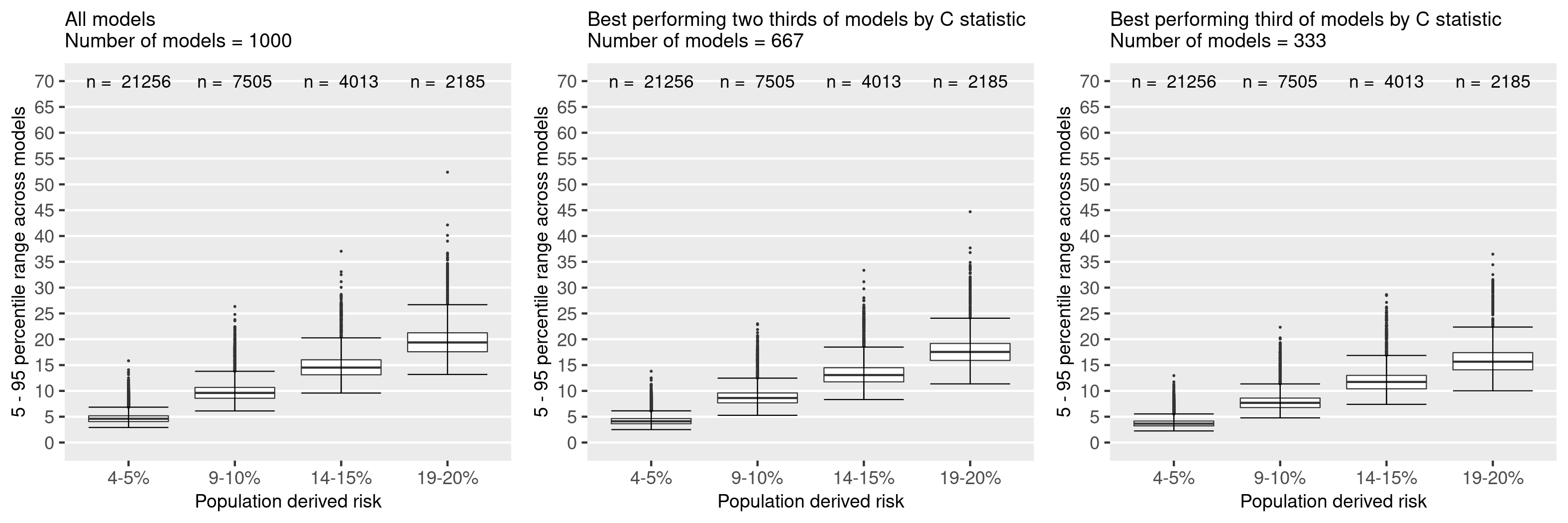


Sample size = 50 000


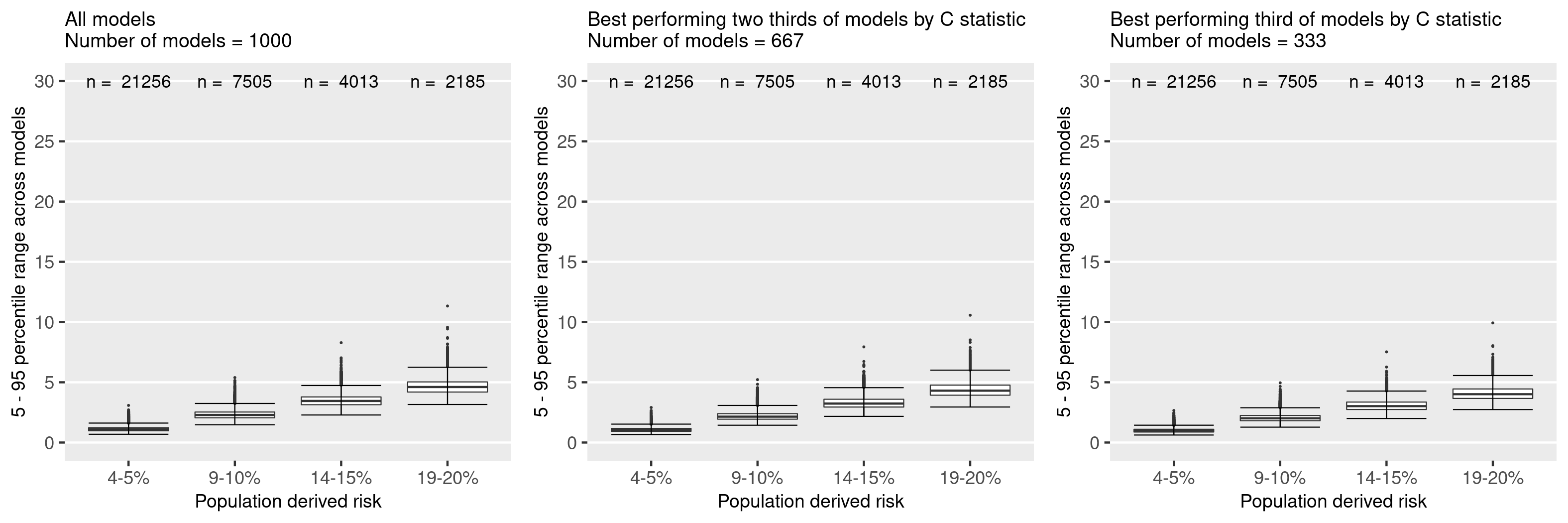


Sample size = 100 000


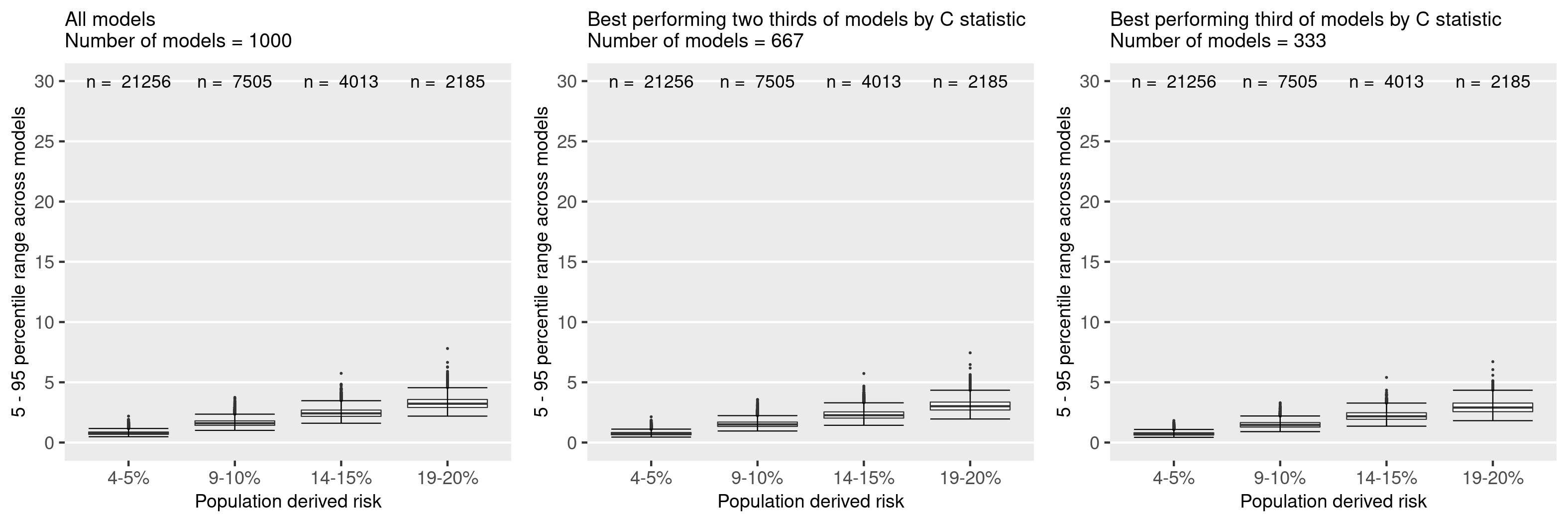


# Supplementary Figure 3: Boxplots of the 5 - 95 percentile ranges in risk for individuals across the models, subsetted by the C statistic of the models (male cohort)

Sample size = N_min_ (1405)


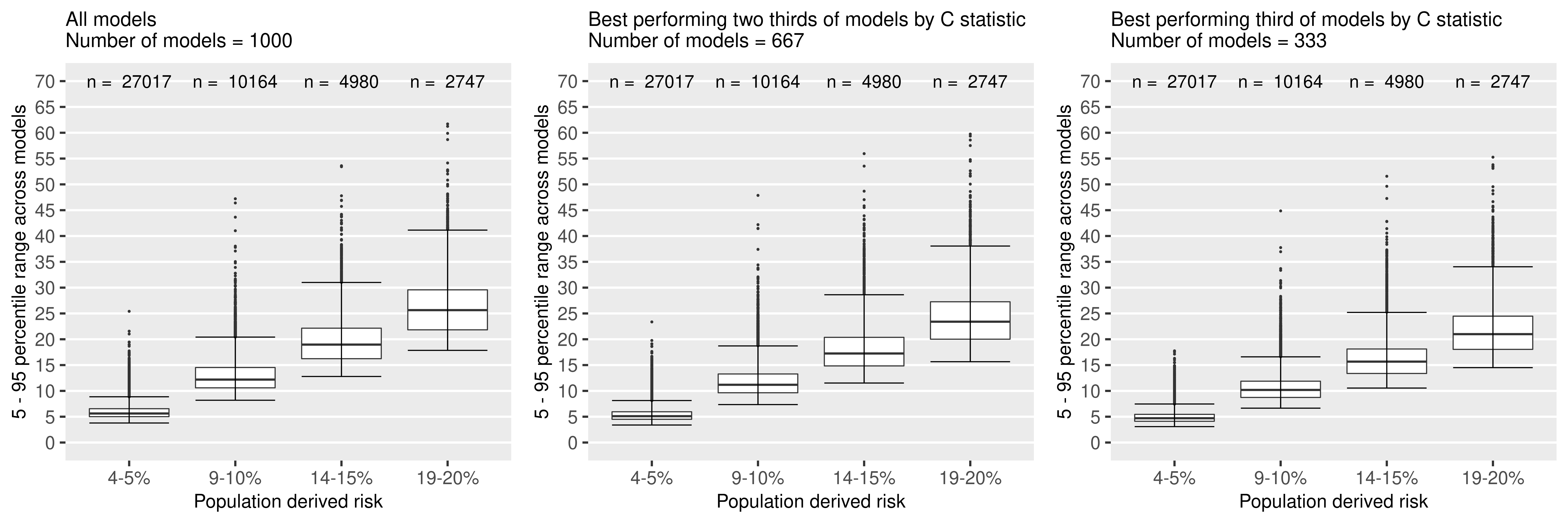


Sample size = N_epv10_ (2297)


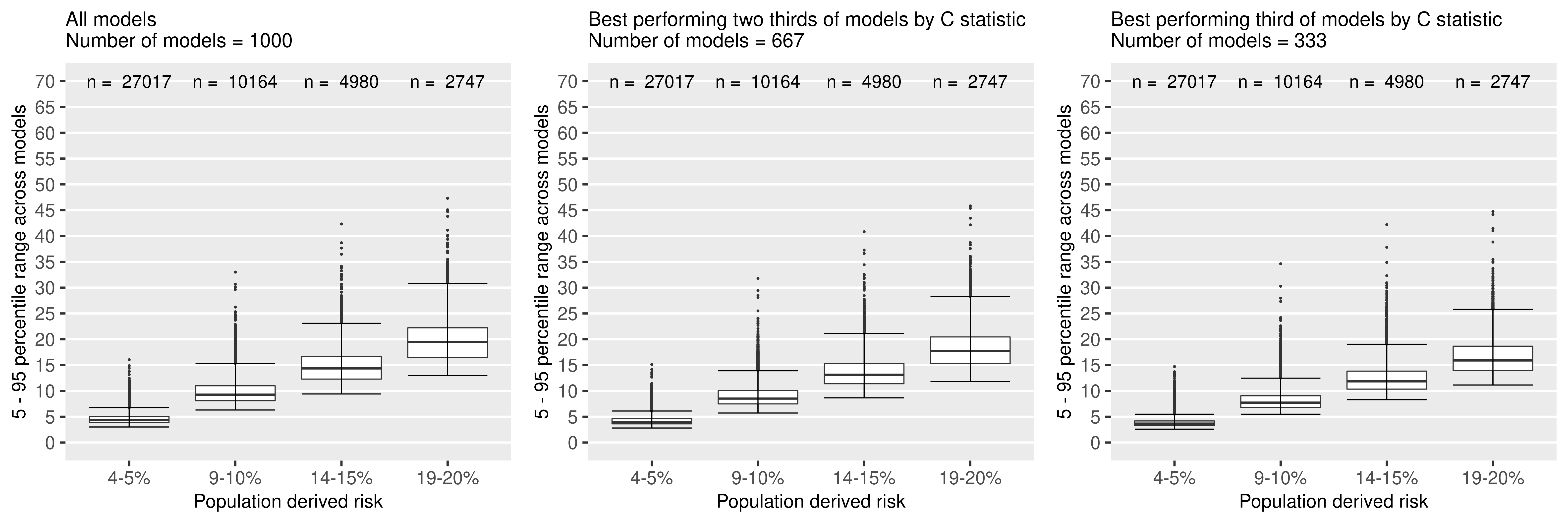


Sample size = 10 000


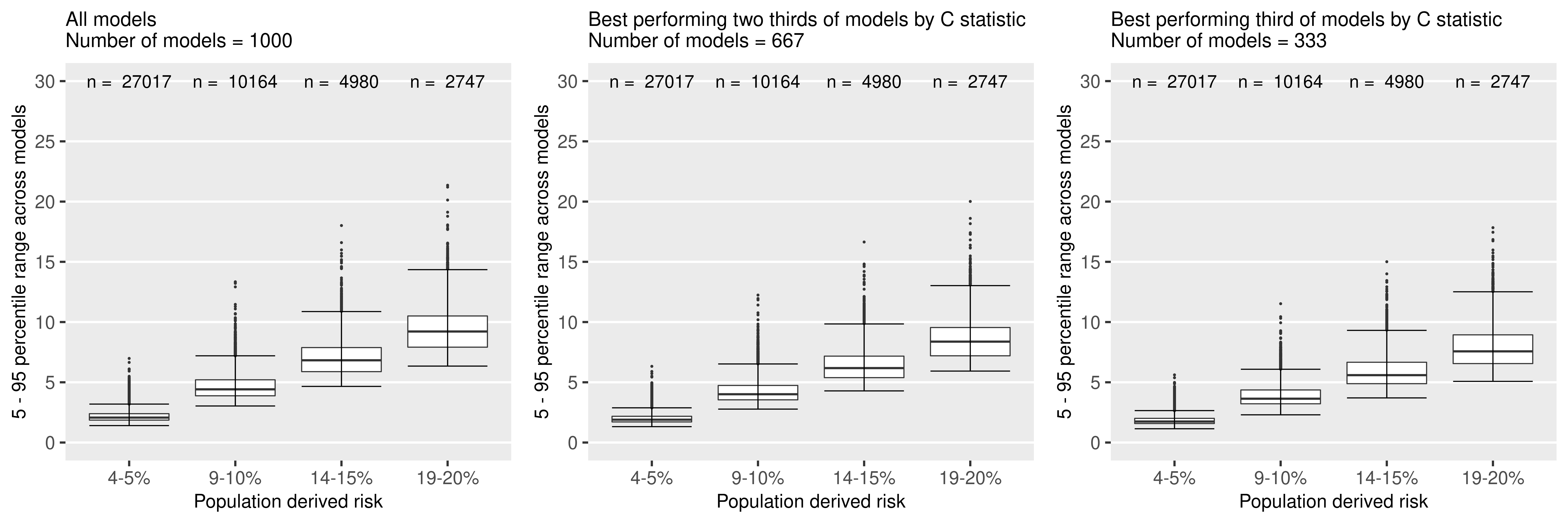


Sample size = 50 000


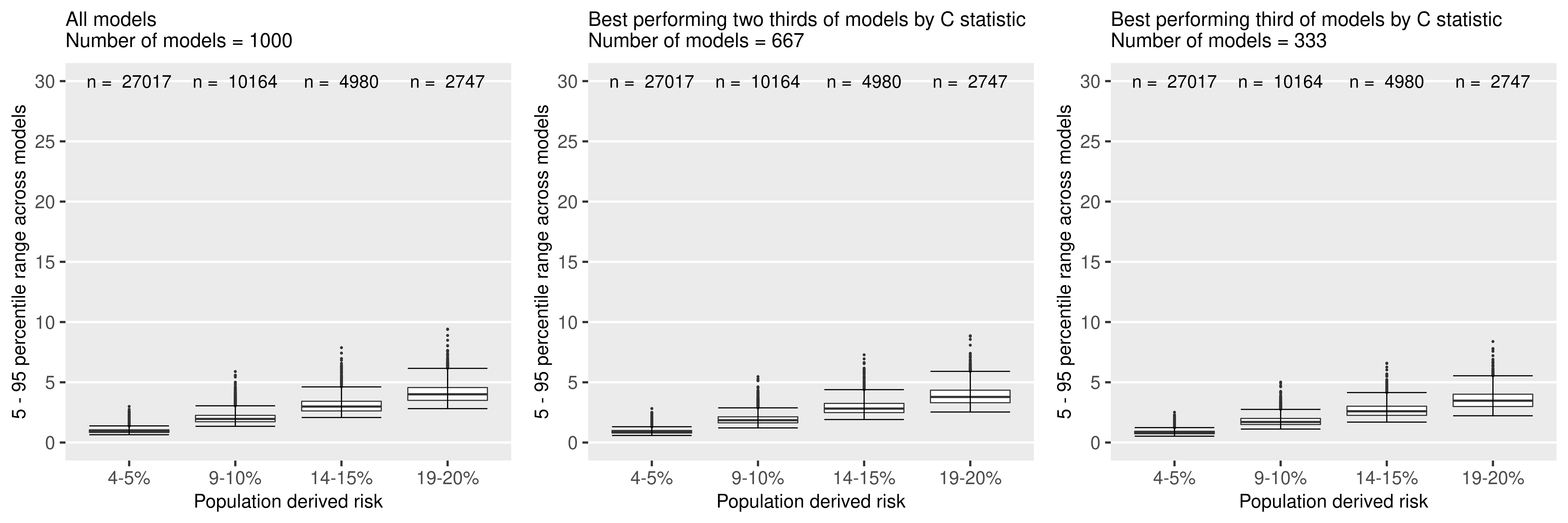


Sample size = 100 000


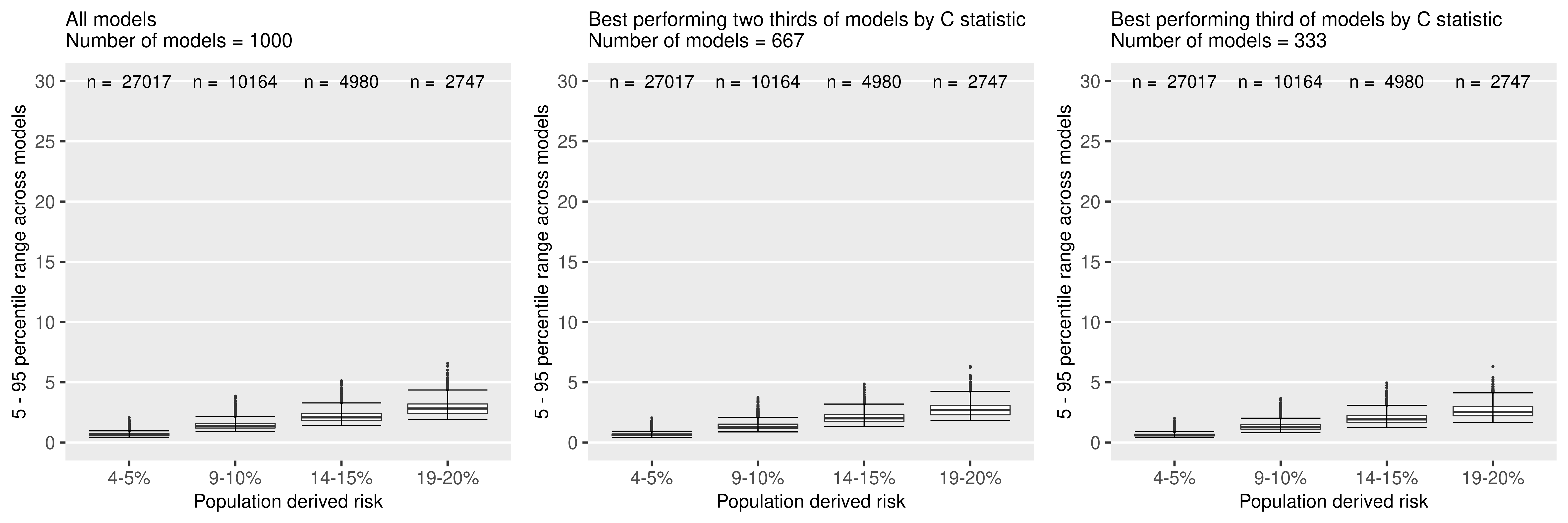


# Supplementary Figure 4: Boxplots of the 5 - 95 percentile ranges in risk for individuals across the models, subsetted by the calibration-in-the-large of the models (female cohort)

Sample size = N_min_ (1434)


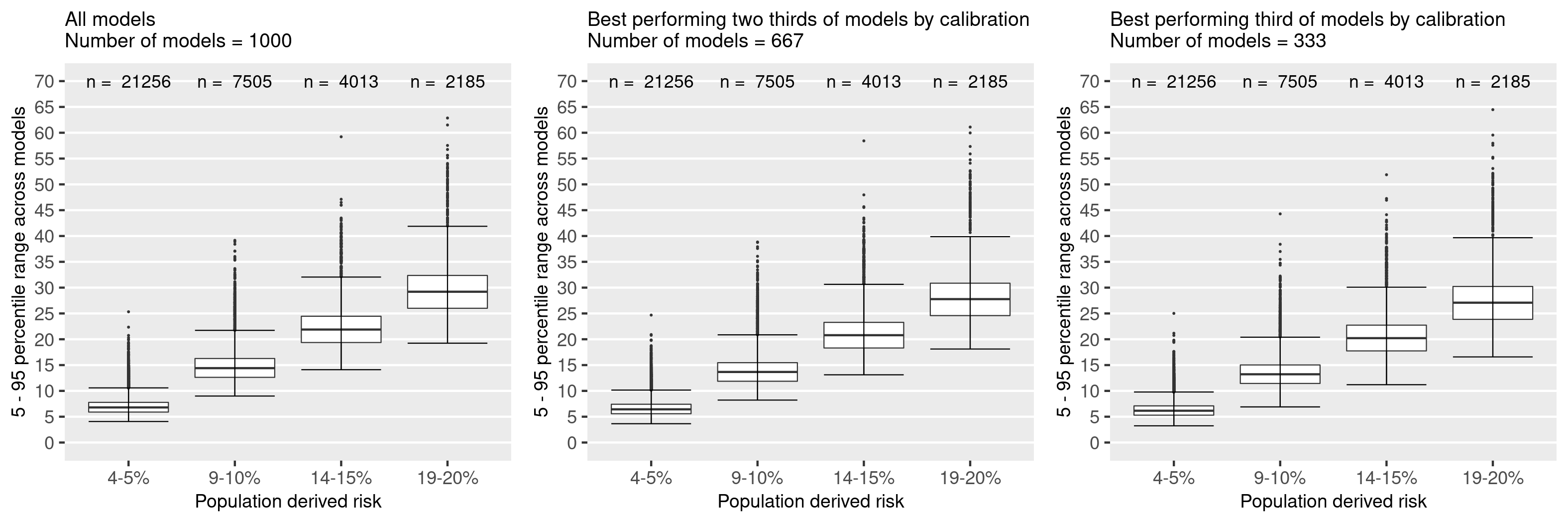


Sample size = N_epv10_ (2954)


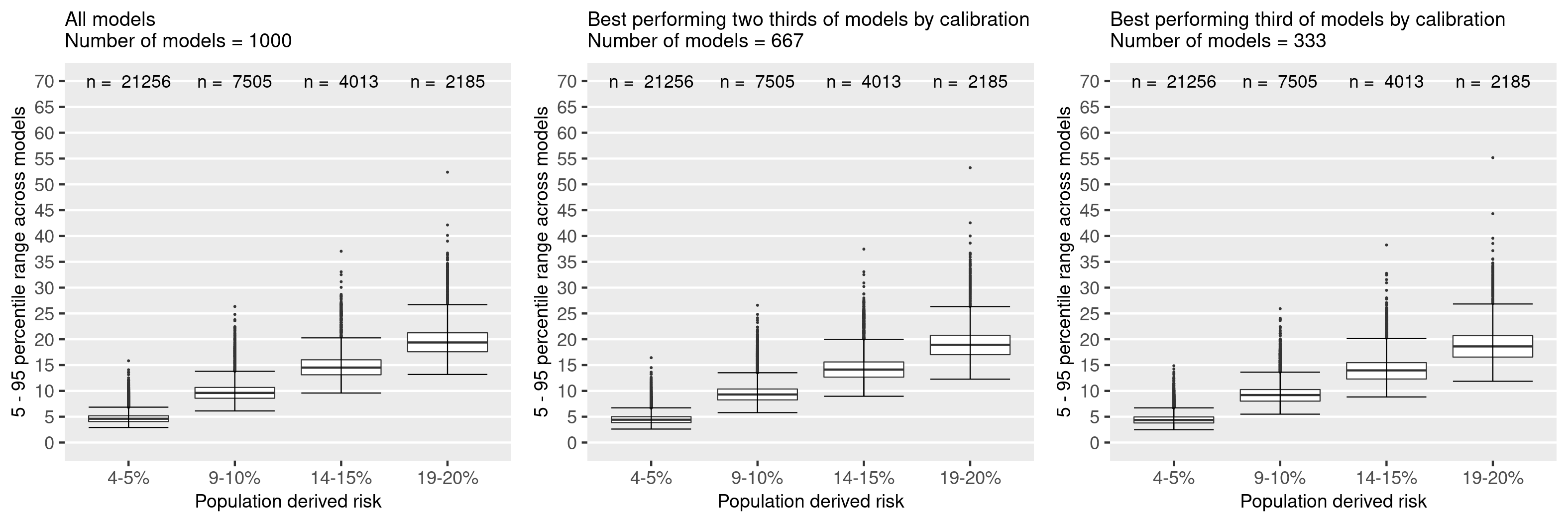


Sample size = 50 000


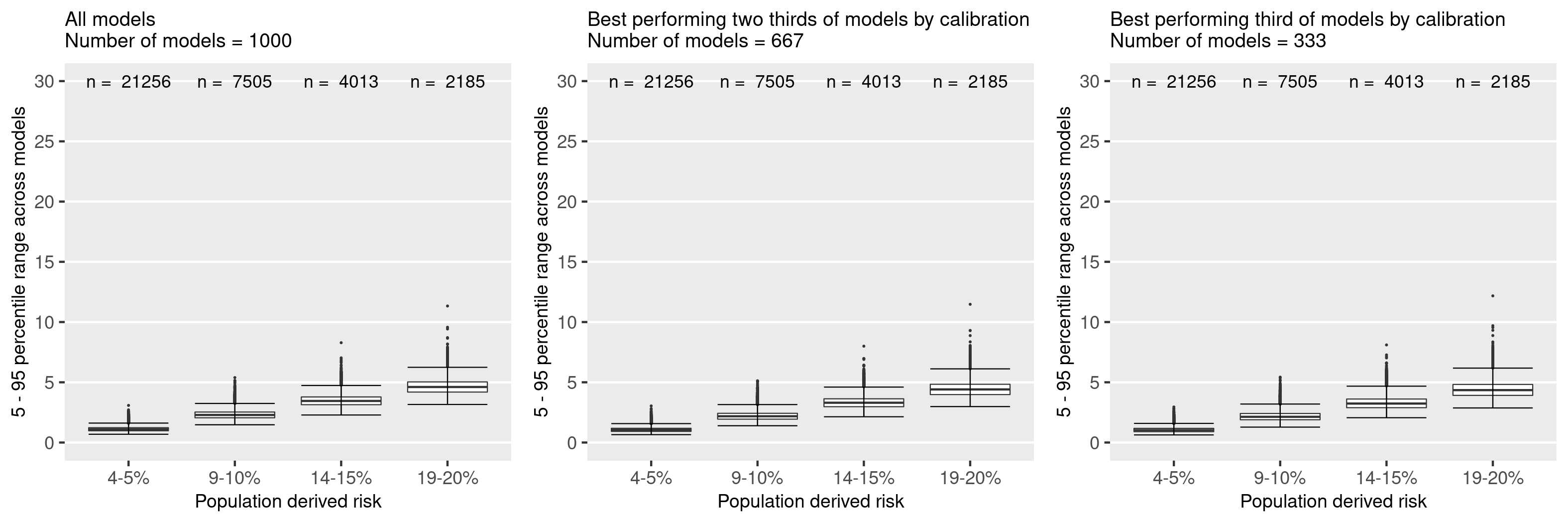


Sample size = 100 000


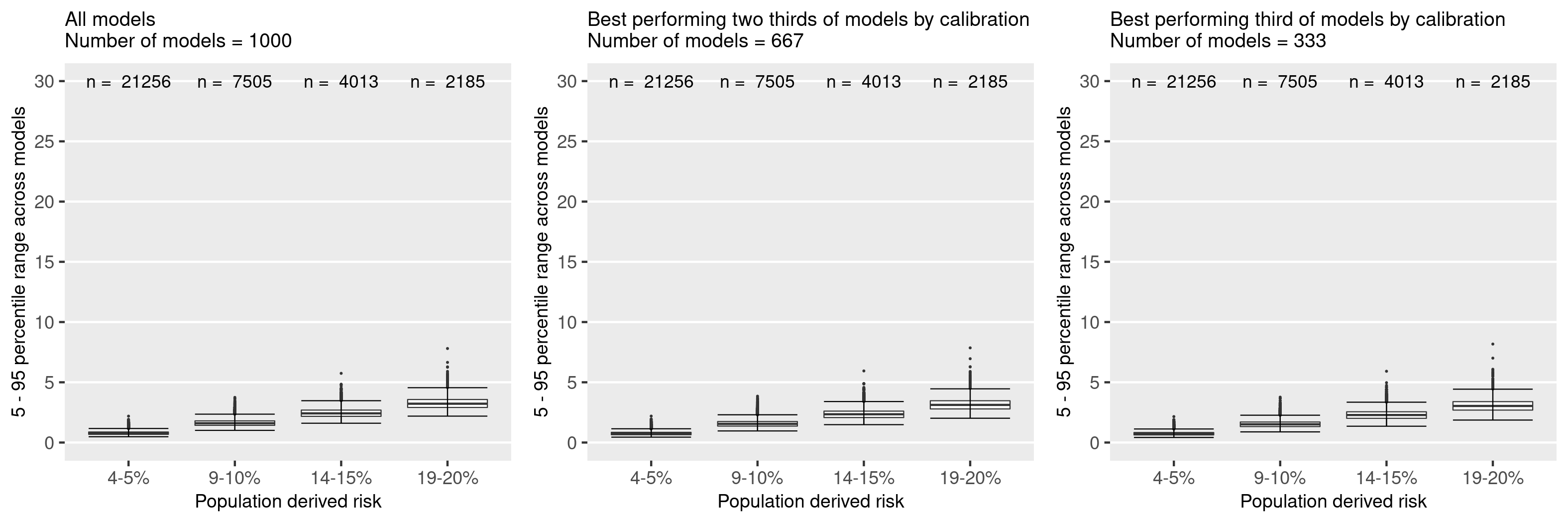


# Supplementary Figure 5: Boxplots of the 5 - 95 percentile ranges in risk for individuals across the models, subsetted by the calibration-in-the-large of the models (male cohort)

Sample size = N_min_ (1405)


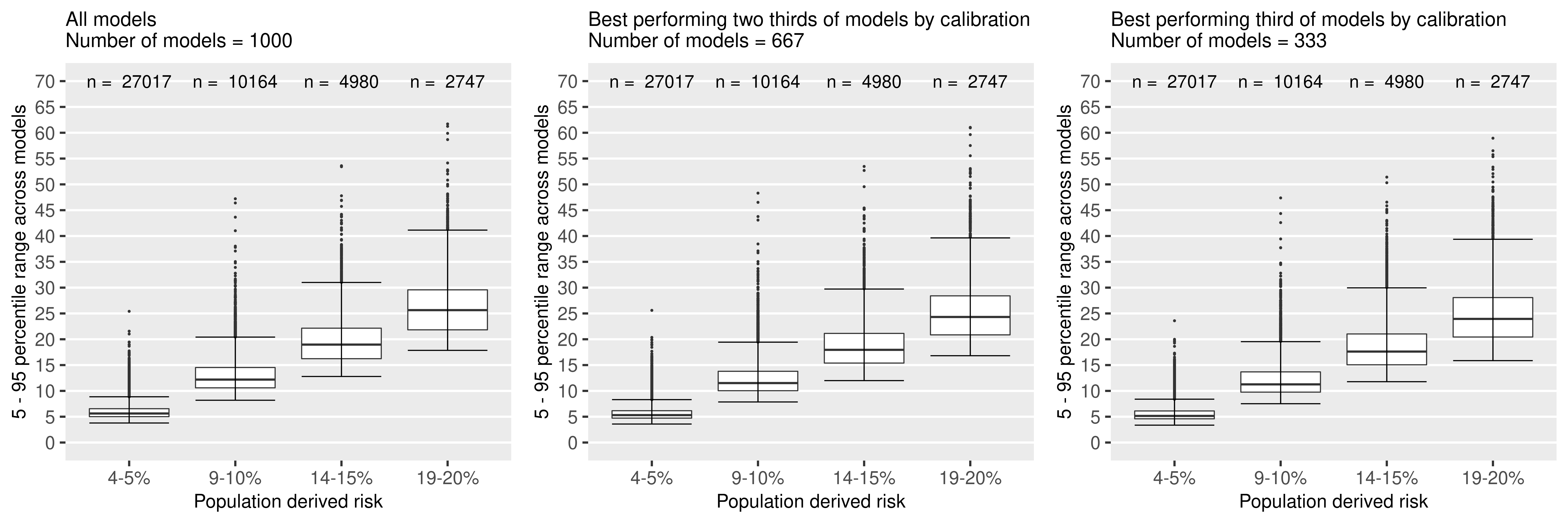


Sample size = N_epv10_  (2297)


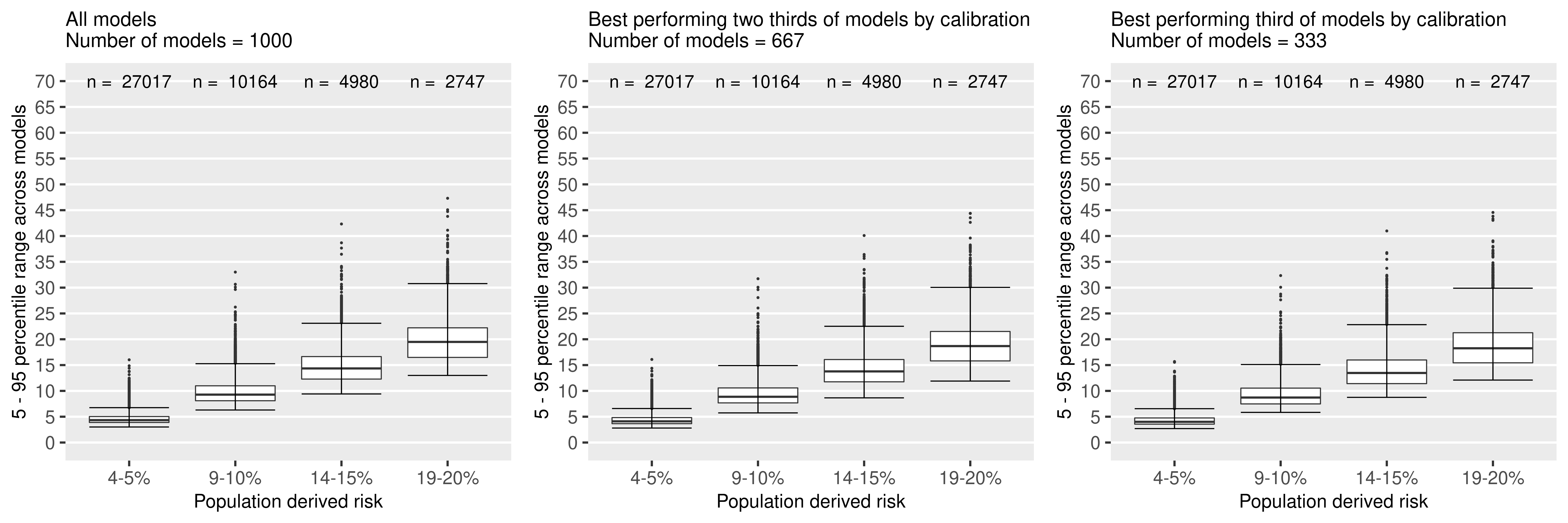


Sample size = 10 000


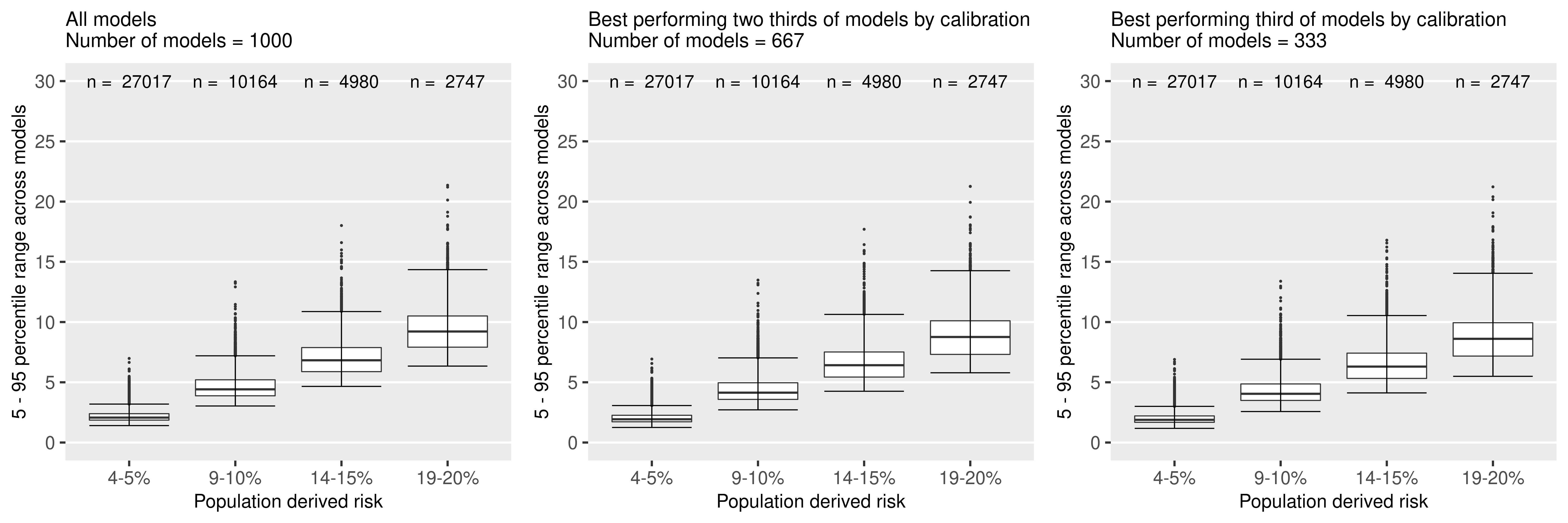


Sample size = 50 000


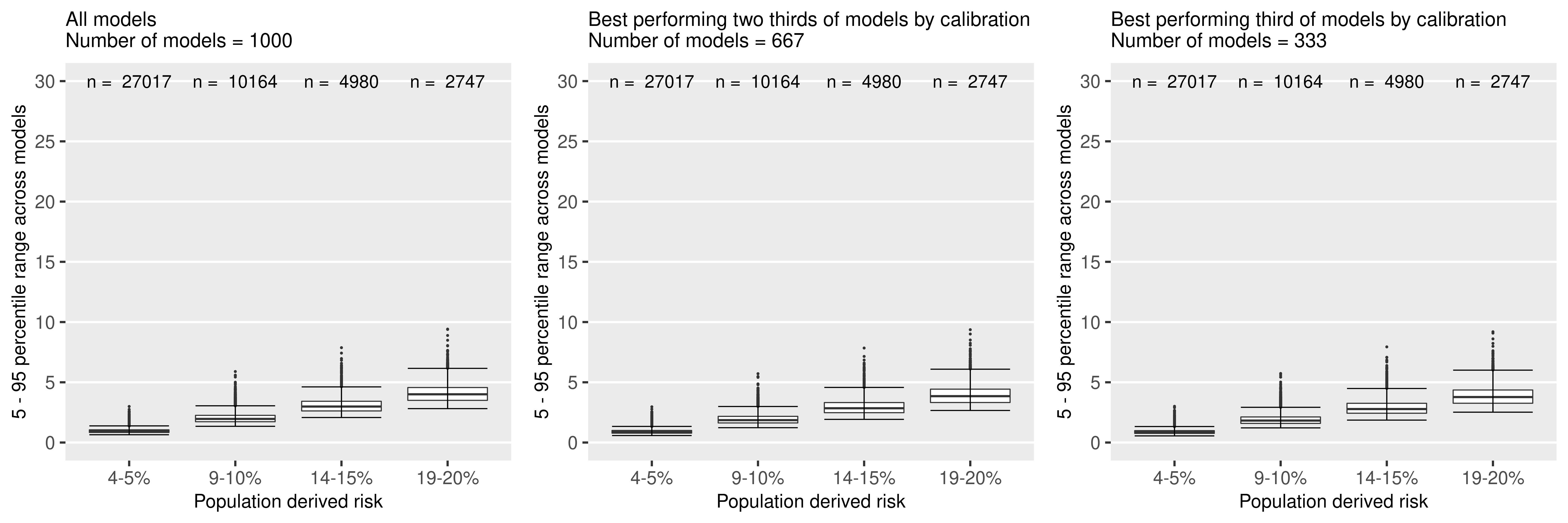


Sample size = 100 000


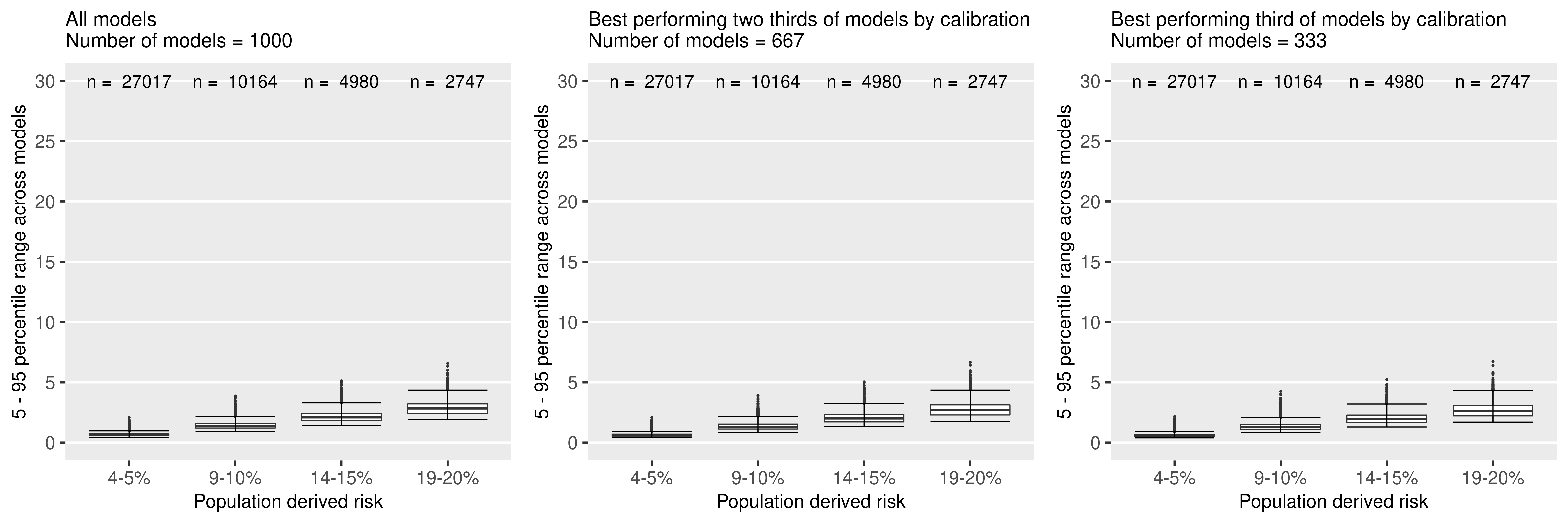


# Supplementary Figure 6: Boxplots of the 5 - 95 percentile ranges in risk for individuals across the models, subsetted by the MAPE_practical_ of the models (female cohort)

Sample size = N_min_ (1434)


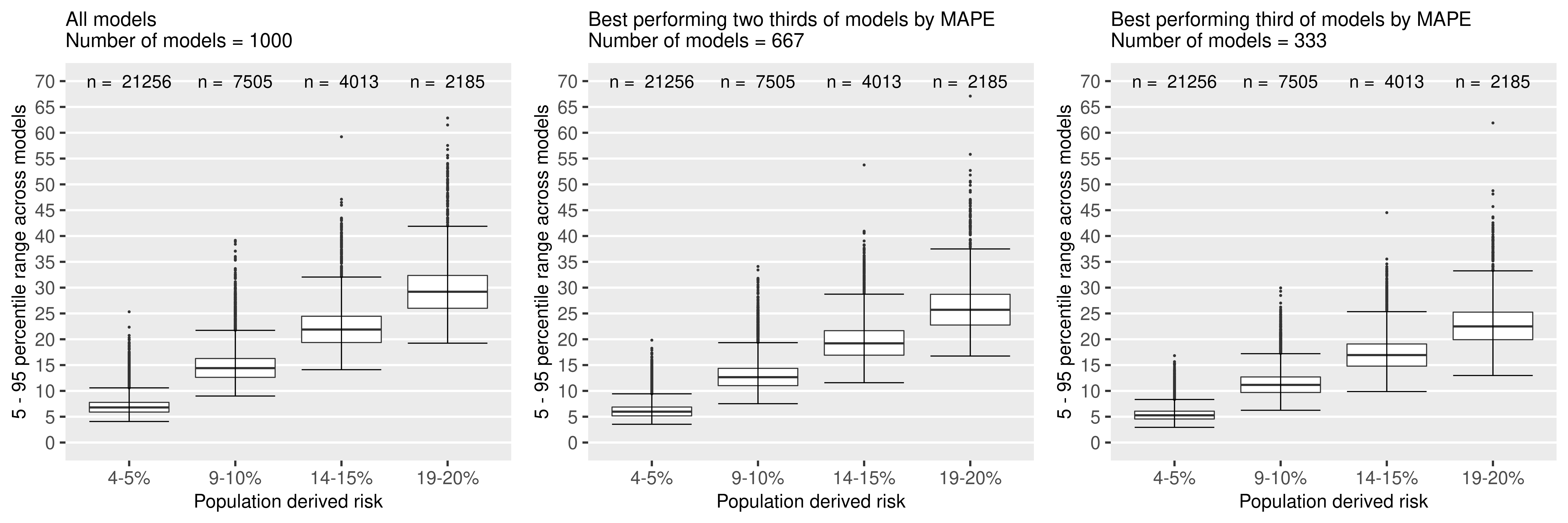


Sample size = N_epv10_ (2954)


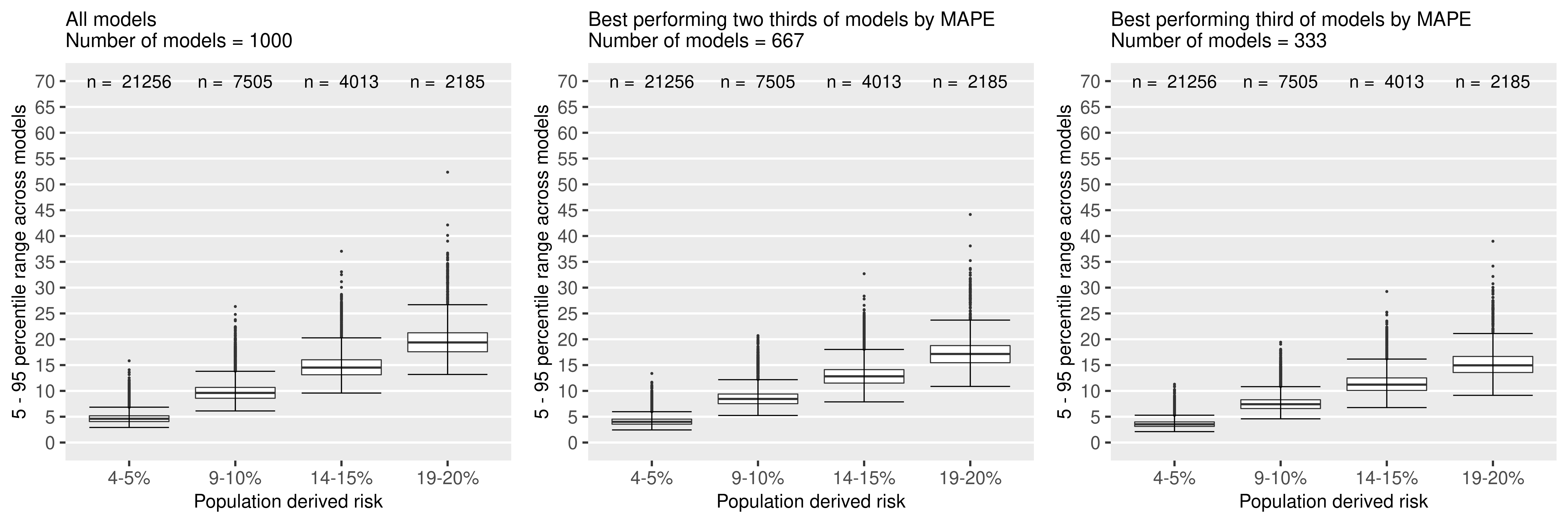


Sample size = 50 000


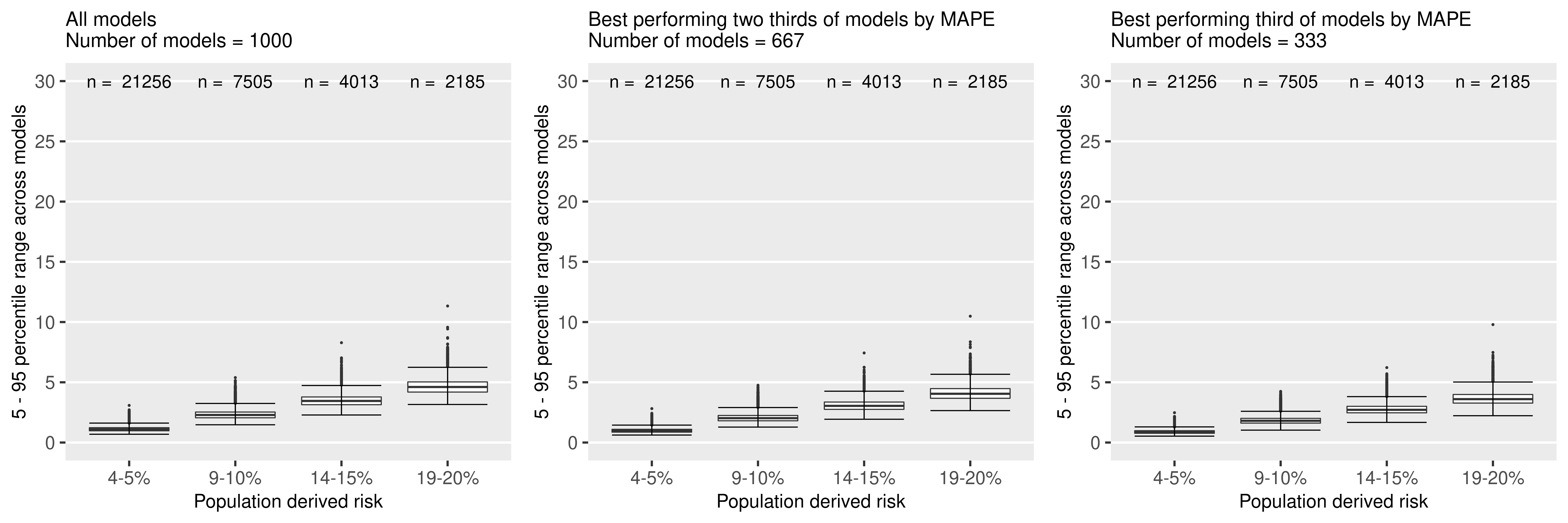


Sample size = 100 000


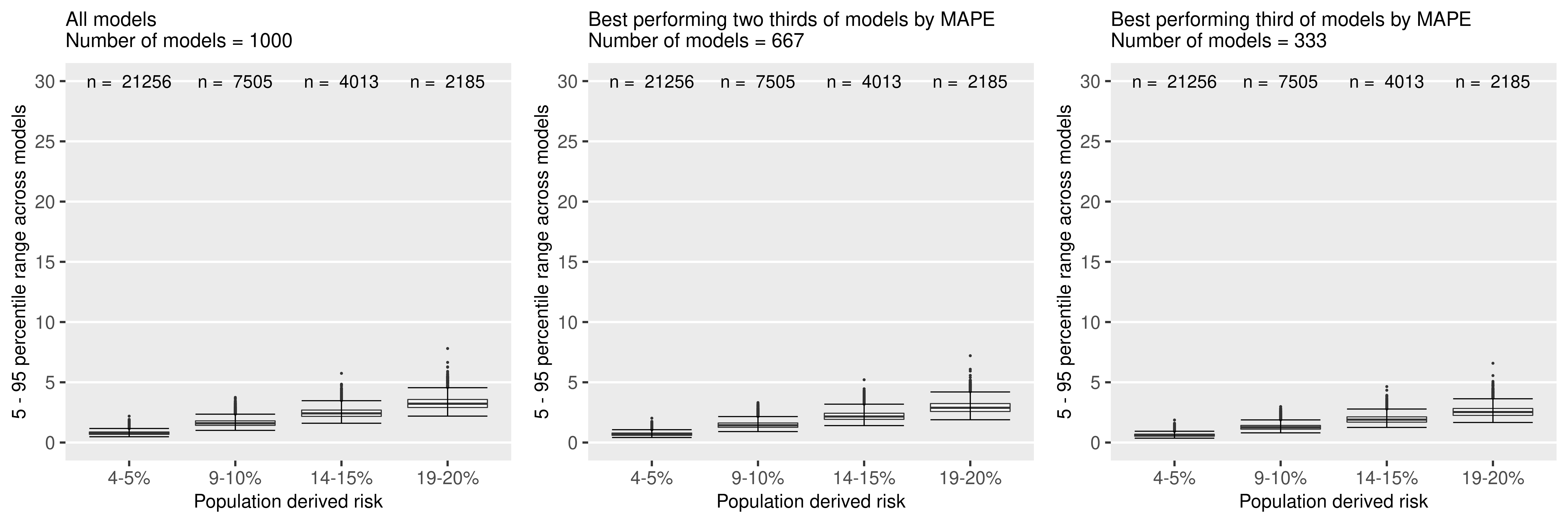


# Supplementary Figure 7: Boxplots of the 5 - 95 percentile ranges in risk for individuals across the models, subsetted by the MAPE_practical_ of the models (male cohort)

Sample size = N_min_ (1405)


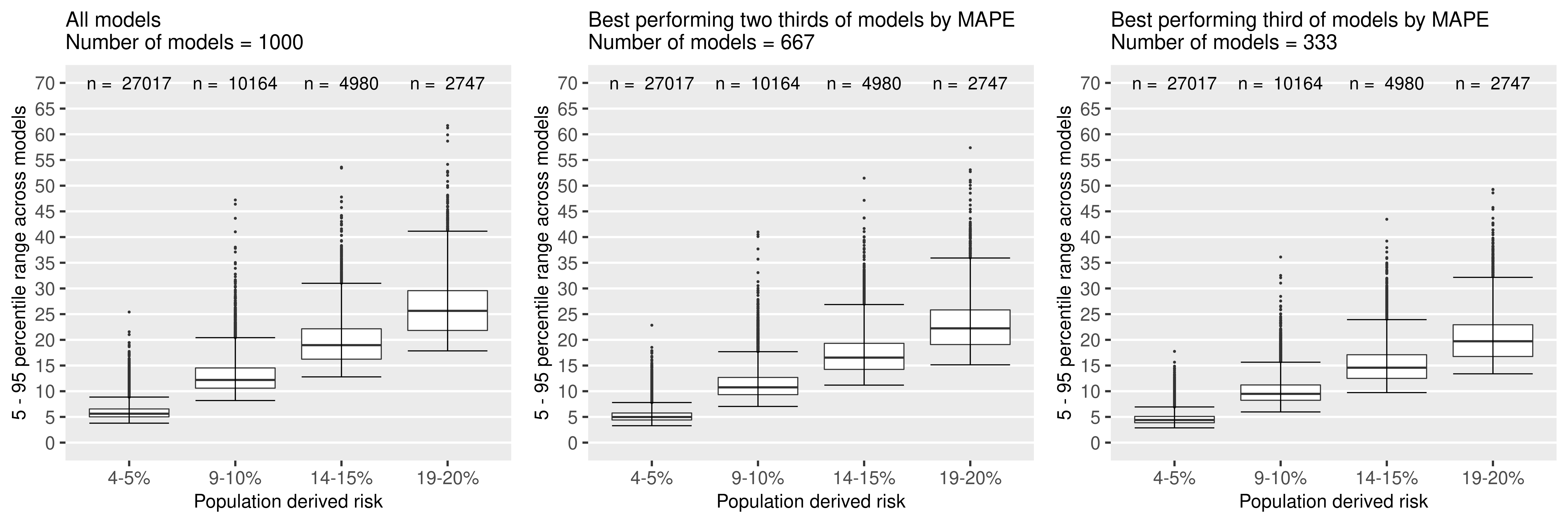


Sample size = N_epv10_ (2297)


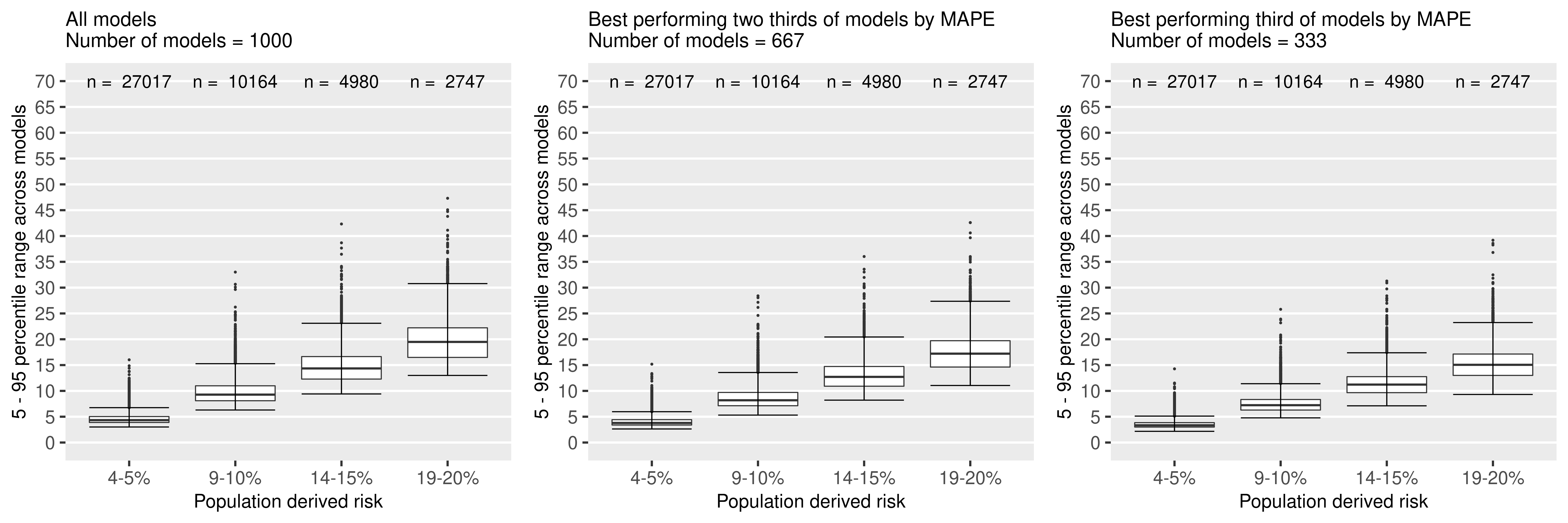


Sample size = 10 000


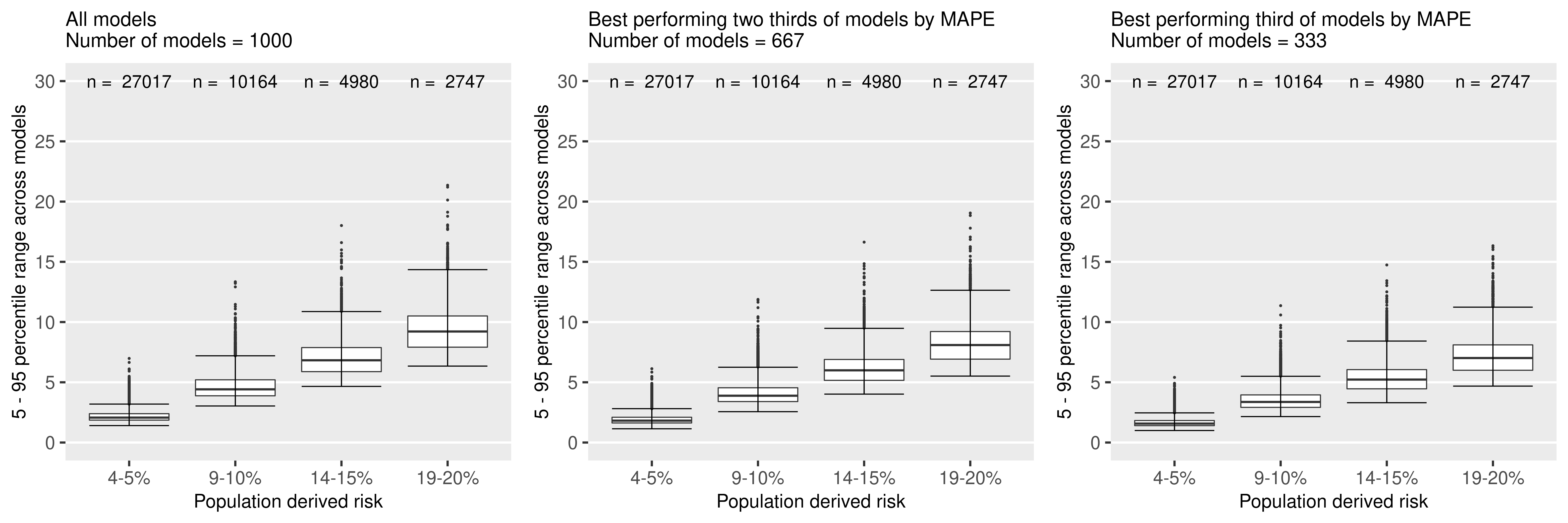


Sample size = 50 000


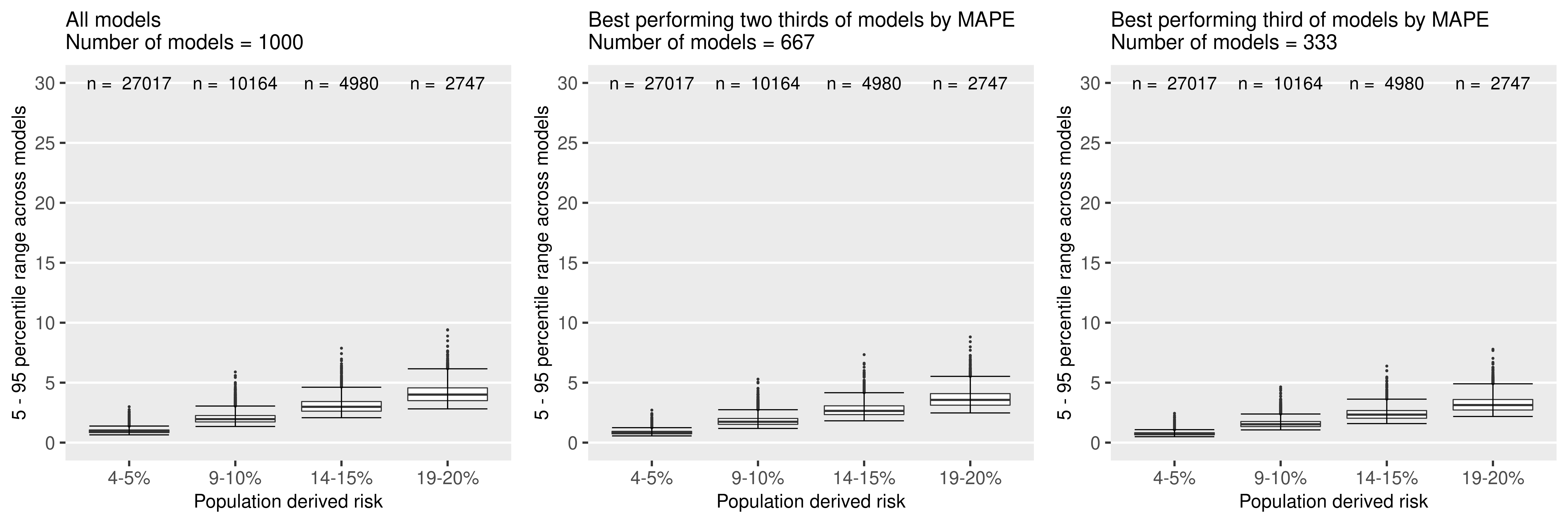


Sample size = 100 000


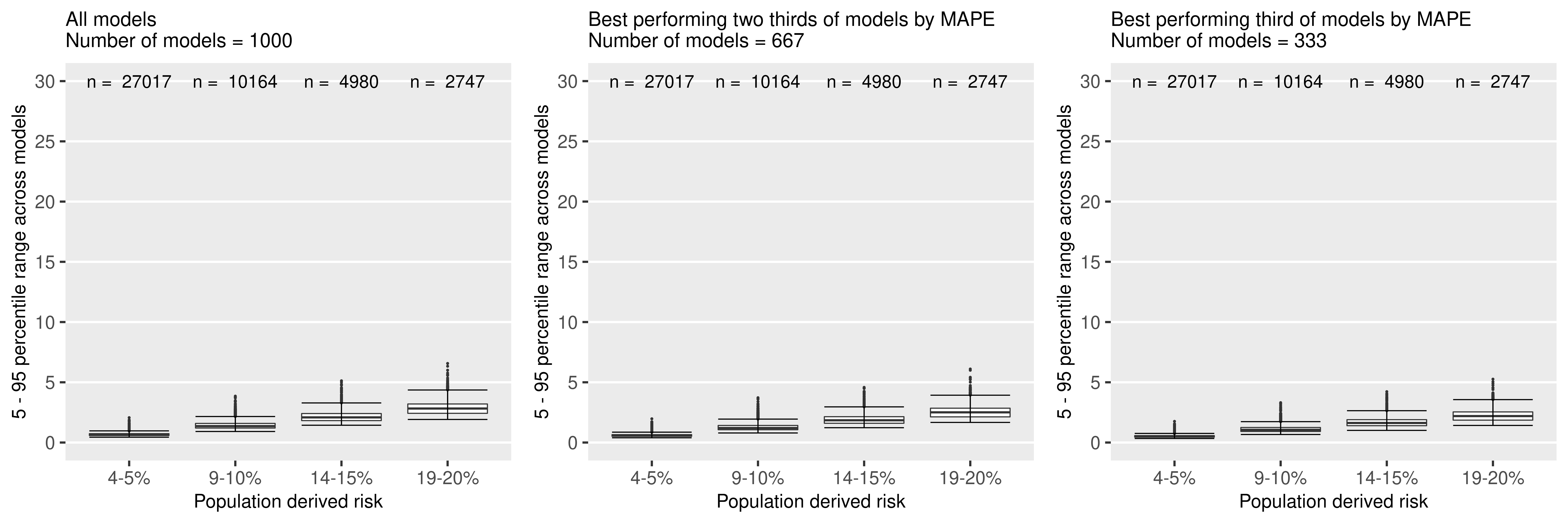

Supplement: Supplementary file 3 — Additional file 3. Supplementary tables and figures. Baseline demographics of male cohorts and results from simulations that could not be included in the main manuscript for space reasons [file 41512_2020_82_MOESM3_ESM.docx]
